# Acetate functions as an epigenetic metabolite to promote lipid synthesis under hypoxia

**DOI:** 10.1038/ncomms11960

**Published:** 2016-06-30

**Authors:** Xue Gao, Shu-Hai Lin, Feng Ren, Jin-Tao Li, Jia-Jia Chen, Chuan-Bo Yao, Hong-Bin Yang, Shu-Xia Jiang, Guo-Quan Yan, Di Wang, Yi Wang, Ying Liu, Zongwei Cai, Ying-Ying Xu, Jing Chen, Wenqiang Yu, Peng-Yuan Yang, Qun-Ying Lei

**Affiliations:** 1 Key Laboratory of Metabolism and Molecular Medicine, Ministry of Education, and Department of Biochemistry and Molecular Biology, School of Basic Medical Sciences, and Institutes of Biomedical Sciences, Fudan University, Shanghai 200032, China; 2 School of Life Science, Fudan University, Shanghai 200433, China; 3 Department of Biochemistry and Molecular Cell Biology, Shanghai Key Laboratory for Tumor Microenvironment and Inflammation, Shanghai Jiao Tong University School of Medicine (SJTU-SM), Shanghai 200025, China; 4 State Key Laboratory of Environmental and Biological Analysis, Department of Chemistry, Hong Kong Baptist University, Hong Kong 999077, China; 5 Department of Chemistry, Fudan University, Shanghai 200032, China; 6 Department of Pathology, School of Basic Medical Sciences, Fudan University, Shanghai 200032, China; 7 Department of Hematology and Medical Oncology, Winship Cancer Institute of Emory, Emory University School of Medicine, Atlanta, Georgia 30322, USA

## Abstract

Besides the conventional carbon sources, acetyl-CoA has recently been shown to be generated from acetate in various types of cancers, where it promotes lipid synthesis and tumour growth. The underlying mechanism, however, remains largely unknown. We find that acetate induces a hyperacetylated state of histone H3 in hypoxic cells. Acetate predominately activates lipogenic genes *ACACA* and *FASN* expression by increasing H3K9, H3K27 and H3K56 acetylation levels at their promoter regions, thus enhancing *de novo* lipid synthesis, which combines with its function as the metabolic precursor for fatty acid synthesis. Acetyl-CoA synthetases (ACSS1, ACSS2) are involved in this acetate-mediated epigenetic regulation. More importantly, human hepatocellular carcinoma with high ACSS1/2 expression exhibit increased histone H3 acetylation and FASN expression. Taken together, this study demonstrates that acetate, in addition to its ability to induce fatty acid synthesis as an immediate metabolic precursor, also functions as an epigenetic metabolite to promote cancer cell survival under hypoxic stress.

## Abstract (web summary)

Cancer cells under stress use acetate to maintain the acetyl-CoA pool and fuel lipid biosynthesis. Here, the authors show that acetate also promotes *de novo* lipid synthesis by increasing histone acetylation at the promoters of lipogenic enzymes *ACACA* and *FASN*, thus inducing their expression.

## Graphical abstract

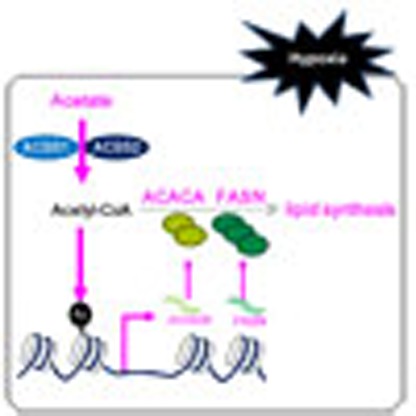

Acetyl-CoA, as a central metabolic intermediate, is widely used in macromolecule biosynthesis and energy production to support cell growth and proliferation. As a donor of acetyl group, acetyl-CoA is also dynamically associated with acetylation modification to modulate protein functions. Therefore, maintenance of cellular acetyl-CoA pool is essential for the regulation of various cellular processes.

In human, acetyl-CoA is mainly produced from oxidation of glucose and other conventional carbon sources, such as glutamine and fatty acids^1^,^2^. However, in human brain cancers, glucose contributes <50% carbons to cellular acetyl-CoA pool, suggesting the existence of a substitutive supply for acetyl-CoA^3^. Subsequent studies reveal that cancer cells avidly capture acetate as their alternative carbon source to support cell survival and proliferation under stressed conditions, in particular hypoxia^3^,^4^,^5^,^6^,^7^,^8^,^9^. Moreover, various human cancers show enhanced acetate uptake in [^11^C]-acetate PET studies^10^,^11^,^12^,^13^,^14^,^15^. These findings suggest that cancer cells utilize acetate as an alternative carbon source to glucose to maintain cellular acetyl-CoA pool under stressed conditions.

Acetate has long been identified as a major carbon source in bacteria and yeasts. Yeast acetyl-CoA synthetases (Acs1p and Acs2p) fuel cell growth by converting acetate to acetyl-CoA^16^. Very recently, acetate is also found to be an alternative carbon source besides glucose, glutamine and fatty acids in human cancer, attracting intensive investigations^7^,^8^. Generally, mammalian acetyl-CoA synthesis from acetate is carried out by ACSS2 to support lipid synthesis in the cytosol, and by ACSS1 to fuel ATP production in mitochondria^17^,^18^,^19^. Acetate is mainly acquired from diet, but can also be generated in ethanol metabolism or deacetylation processes. The function of acetate has long been overlooked due to its relative low physiological concentration (0.2–0.3 mM) in blood^20^. Recent studies reveal that cancer cells show increased acetate uptake under hypoxia even in the presence of low acetate concentration to support tumour growth^7^,^9^,^14^. However, how cancer cells utilize acetate under hypoxia in such an efficient manner remains unclear.

Histone acetylation is intimately coordinated with cellular acetyl-CoA pool in response to metabolic state. As the downstream metabolite of carbon sources, acetyl-CoA represents a pivotal metabolic signal of nutrient availability^4^,^21^,^22^. In yeast, histone is specifically acetylated at genes involved in lipogenesis, aminoacid biosynthesis and cell cycle progression upon entry into growth, in tune with intracellular acetyl-CoA level^23^. ATP citrate lyase (ACLY), the enzyme converting glucose-derived citrate into acetyl-CoA, regulates histone acetylation by sensing glucose availability^1^,^22^. Yeast acetyl-CoA Carboxylase (Acc1p) consumes acetyl-CoA to synthesize lipids and regulates global histone acetylation through competing for the same nucleocytosolic acetyl-CoA pool^24^. Thus, the acetyl-CoA flux dynamically regulates gene expression profile by modulating histone acetylation state.

These observations led us to hypothesize that acetate induces a metabolic adaptation through modulating histone acetylation in hypoxic cancer cells. Consistent with this idea, we found that acetate predominately activates the expression of lipogenic genes through upregulating histone acetylation at their promoter regions, which in turn promotes lipid synthesis under hypoxia. Beyond a carbon source for macromolecular biosynthesis, our findings highlight an epigenetic role for acetate in metabolic adaptation of cancer cells to hypoxic stress.

## Results

### Acetate restores histone acetylation under hypoxia

**Figure 1:**
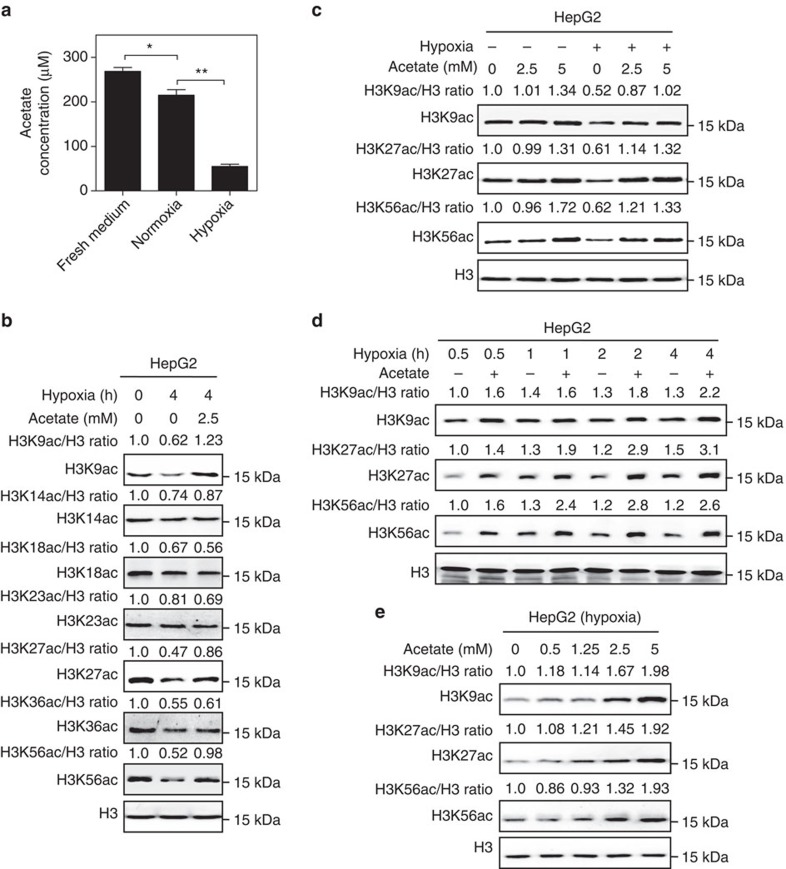
Acetate rescues hypoxia-induced reduction of histone acetylation. (**a**) ^1^H-NMR spectra of acetate concentration in fresh medium, medium of cancer cells cultured under normoxia or hypoxia. The results represented mean±s.d. of triplicate experiments. (^**^P<0.01; *P<0.05; by two-tailed unpaired Student's *t*-test). (**b**) Acetate rescues hypoxia-reduced H3K9, H3K27 and H3K56 acetylation levels. HepG2 cells were treated with or without 2.5 mM acetate under hypoxia (1% O_2_) for 4 h. The histone acetylation levels were determined by western blot. Total H3 served as a loading control. (**c**) Cancer cells are more sensitive to acetate under hypoxia. HepG2 cells were treated with indicated concentrations of acetate under normoxia or hypoxia (1% O_2_) for 4 h. The histone acetylation levels were determined by western blot. Total H3 served as a loading control. (**d**) Acetate increases H3K9, H3K27 and H3K56 acetylation levels in a time-dependent manner under hypoxia. HepG2 cells were treated with or without 5 mM acetate for 0.5, 1, 2 and 4 h under hypoxia (1% O_2_), respectively. The global histone acetylation levels were determined by western blot. Total histone H3 served as a loading control. (**e**) Acetate increases H3K9, H3K27 and H3K56 acetylation levels in a dose-dependent manner under hypoxia. HepG2 cells were treated with the indicated concentrations of acetate for 4 h under hypoxia (1% O_2_). The histone acetylation levels were determined by western blot. Total H3 served as a loading control.

Cancer cells demand distinctive extracellular nutrients and reprogram the metabolic pathways to survive and proliferate when facing harsh situation, such as hypoxia^25^. Hypoxia stress is a critical player in tumorigenesis and tumour development^26^. By performing exometabolome analysis based on ^1^H-NMR spectra, we found that cancer cells absorbed around 20% acetate from the culture medium under normoxia while more than 80% acetate was consumed under hypoxia (Fig. 1a; Supplementary Fig. 1a,b), suggesting that cancer cells take up more acetate under hypoxia than nomoxia^7^,^9^,^14^. Moreover, we carried out the quantification of acetate from five pairs of hepatocellular carcinoma (HCC) and adjacent samples by NMR. As shown in Supplementary Fig. 1c, acetate concentration range was from 0.56 to 2.67 μmol g^−1^ in wet tissue (left) and acetate concentration in tissue would be roughly estimated around from 0.56 to 2.67 mM (right). In most cases acetate concentrations ranged around 0.5 mM and in two HCC samples acetate levels reached 2.5 mM (Supplementary Fig. 1c). To explore acetate effect on cancer cell, we treated HepG2 cells with acetate under hypoxia and found that acetate counteracted the declined histone acetylation under hypoxia compared with normoxia (Fig. 1b). Of particular interest, acetate induced significant increase of H3K9, H3K27 and H3K56 acetylation levels, but not H3K14, H3K18, H3K23 and H3K36 acetylation levels (Fig. 1b). This indicates that acetate rescues hypoxia-reduced histone acetylation with certain specificity. More importantly, by using metabolic-labelling technique based on liquid chromatography tandem mass spectrometry in multiple reaction monitoring mode (LC-MRM MS), we identified ^13^C_2_-labelled acetylated H3K9 (Supplementary Fig. 1d), H3K27 (Supplementary Fig. 1e) and H3K56 (Supplementary Fig. 1f) peptides in HepG2 cells treated with [U-^13^C_2_]-acetate, demonstrating that acetate-derived acetyl-CoA was indeed incorporated into histones. In addition, compared with normoxia, histone acetylation is more susceptible to acetate supplementation under hypoxia, even under low concentration, in HepG2, A549 and DU 145 cells (Fig. 1c; Supplementary Fig. 1g,h).

To characterize the manner of epigenetic regulation by acetate, we treated cancer cells with acetate for different time points under hypoxia. We found that exogenous acetate supplement rapidly increased H3K9, H3K27 and H3K56 acetylation levels in a time-dependent manner under hypoxia in different cancer cell lines (Fig. 1d; Supplementary Fig. 1i,j). Acetate supplement dramatically increased H3K9, H3K27 and H3K56 acetylation levels within 2 h (Fig. 1d; Supplementary Fig. 1i,j). Furthermore, acetate was found to increase histone H3K9, H3K27 and H3K56 acetylation levels in a dose-dependent manner under hypoxia in HepG2 (Fig. 1e), A549 (Supplementary Fig. 1k), DU145 (Supplementary Fig. 1l), SkBr3 (Supplementary Fig. 1m) and HT29 cells (Supplementary Fig. 1n). Low concentration of acetate (1.25 mM) slightly increased histone acetylation levels, while higher concentrations of acetate (2.5–5 mM) significantly increased histone acetylation levels under hypoxia (Fig. 1e; Supplementary Fig. 1k, l, m, n). Collectively, these data imply that acetate is implicated in the regulation of histone acetylation under hypoxia.

### Acetate epigenetically activates de novo lipogenesis

**Figure 2:**
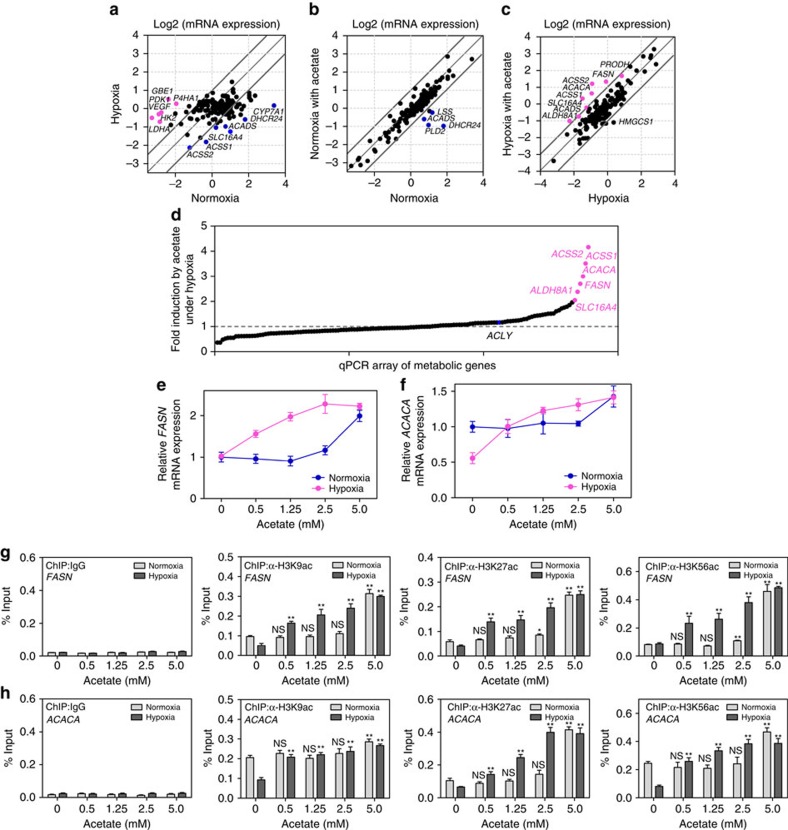
Acetate predominately activates lipid synthesis pathway through epigenetic regulation under hypoxia. (**a**) Scatter plot of mRNA expression data of 139 metabolic genes in HepG2 cells, comparing cells treated with hypoxia (*y* axis) to normoxia (*x* axis). The mRNA expression value of triplicate experiments was shown on a log 2 scale. Grey lines indicated two-fold differences in the mRNA expression levels between two groups. Upregulated genes (>2-fold change, *P*<0.05) were shown in magenta. Downregulated expressed genes (>2-fold change, *P*<0.05) were shown in blue. Two-tailed unpaired Student's *t*-test was used. (**b**) Scatter plot of mRNA expression data of 139 metabolic genes in HepG2 cells under normoxia, comparing cells treated with 2.5 mM acetate (*y* axis) to acetate-free (*x* axis). The mRNA expression value of triplicate experiments was shown on a log2 scale. Grey lines indicated two-fold differences in the mRNA expression levels between two groups. Downregulated genes (>2-fold change, *P*<0.05) were shown in blue. Two-tailed unpaired Student's *t*-test was used. (**c**) Scatter plot of mRNA expression data of 139 metabolic genes in HepG2 cells under hypoxia, comparing cells treated with 2.5 mM acetate (*y* axis) with acetate-free (*x* axis). The mRNA expression value of triplicate experiments was shown on a log 2 scale. Grey lines indicated two-fold differences in the mRNA expression levels between two groups. Upregulated genes (>2-fold change, *P*< 0.05) were shown in magenta. Two-tailed unpaired Student's *t*-test was used. (**d**) Fold-change analysis of the mRNA expression of 139 genes in HepG2 cells treated with or without 2.5 mM acetate under hypoxia. (**e**,**f**) *FASN* (**e**) and *ACACA* (**f**) mRNA levels in HepG2 cells treated with indicated concentrations of acetate for 12 h under normoxia or hypoxia were quantified by qPCR. The results were presented as mean±s.d. of triplicate experiments. (**g**,**h**) ChIP-qPCR assays showing H3K9, H3K27 and H3K56 acetylation enrichment at *FASN* (**g**) and *ACACA* (**h**) promoter regions in HepG2 cells treated with indicated concentrations of acetate under normoxia or hypoxia for 4 h. Rabbit IgG was included as a negative control. Each histogram was presented as mean±s.d. of triplicate experiments (*P<0.05; ^**^P<0.01; NS, not significant; by two-tailed unpaired Student's *t*-test).

It is well known that histone acetylation is associated with transcription activity^27^,^28^,^29^. To investigate the biological effect on cancer metabolism induced by the acetate-mediated histone acetylation, we detected the mRNA expression changes of metabolic genes. We designed quantitative PCR (qPCR) primers targeting 139 metabolic genes, covering a wide range of metabolic pathways (that is, glycolysis, TCA cycle, pentose phosphate pathway, glycogen metabolism, ketone body metabolism, aminoacid metabolism, fatty acid synthesis and β-oxidation, cholesterol synthesis, sphingolipid metabolism, and metabolism-related transporters) (Supplementary Table 1) and conducted qPCR to screen potential metabolic pathway(s) affected by acetate-induced epigenetic regulation. Scatter plot of mRNA expression data of 139 genes in HepG2 cells was shown in Fig. 2a, by comparing cell culture under hypoxia with normoxia. The expression of *VEGF* and *LDHA*, which were included as positive controls for verifying hypoxic effect, increased by more than two-fold under hypoxia (Fig. 2a). We then tested the effect of acetate on gene expression under either normoxia (Fig. 2b) or hypoxia (Fig. 2c), respectively. Compared with the normoxia group, the upregulated genes by two-fold in hypoxia group and hypoxia with acetate group showed a lot overlap (Supplementary Fig. 2a). Besides, *FASN* and *ACSS2* were the unique genes in hypoxia with acetate group, indicating that acetate played an important role in lipid synthesis under hypoxia (Supplementary Fig. 2a). In line with this, *FASN* and *ACACA* mRNA levels were activated by more than two-fold with acetate under hypoxia, while no activation effect was observed under normoxia (Fig. 2b,c). Both *FASN* and *ACACA* are in the top-10 list of upregulated metabolic genes induced by acetate under hypoxia, indicating that acetate predominantly affects *de novo* lipid synthesis compared with other metabolic pathways (Fig. 2c,d). Interestingly, *ACSS1 and ACSS2* mRNA levels were decreased under hypoxia compared with normoxia (Fig. 2a), which was discrepant with the other studies reported that *ACSS2* was upregulated under hypoxia^9^,^14^. This may be due to the experimental conditions, such as different cell lines used and treating time points. Moreover, mRNA level of *ACSS1* and *ACSS2*, rather than *ACLY*, were also upregulated on acetate treatment under hypoxia (Fig. 2c,d). The rescue of hypoxia-reduced *ACSS1* and *ACSS2* expression by acetate may indicate that ACSS1 and ACSS2 play an important role in cancer cells adapting to hypoxia.

We further investigated whether acetate-induced histone acetylation was associated with acetate-promoted gene transcription on lipogenesis. We treated cancer cells with gradient concentrations of acetate and found that *FASN* and *ACACA* mRNA expression were upregulated in a dose-dependent manner under hypoxia (Fig. 2e,f). Intriguingly, change in *FASN* and *ACACA* mRNA expression are more prone to low concentration of acetate under hypoxia, compared with normoxic control (Fig. 2e,f). To characterize whether the upregulation of lipogenic genes is caused by histone acetylation induced by acetate supplementation, we treated cancer cells with trichostatin A (TSA), a histone deacetylase inhibitor, as a positive control. We found that TSA and acetate increased both the global acetylation level of histone H3 and the mRNA expression of *FASN* and *ACACA*, although TSA and acetate had no synergic effect (Supplementary Fig. 2b). In addition, acetate treatment led to time-dependent enhancement of H3K9, H3K27 and H3K56 acetylation levels (Supplementary Fig. 2c). In line with the increased histone H3 acetylation, *FASN* and *ACACA* mRNA expressions were also elevated in a time-dependent manner (Supplementary Fig. 2c). Furthermore, we carried out ChIP–qPCR assays to define the underlying mechanism of acetate-induced *FASN* and *ACACA* expressions. We found that acetate markedly enhanced H3K56 acetylation level at the promoters of *FASN* and *ACACA* (Supplementary Fig. 2d,e), but not *ACLY* (Supplementary Fig. 2f). Furthermore, we found that histone acetylation (H3K9ac, H3K27ac and H3K56ac) at the promoters of *FASN* and *ACACA* responded to acetate treatment in a dose-dependent manner and histone acetylation was more prone to be induced by low concentration (≤2.5 mM) of acetate under hypoxia, compared with normoxia (Fig. 2g,h). These results demonstrate that acetate is capable of epigenetically regulating lipogenic genes at pathophysiological concentration.

To rule out the possibility that promoter acetylation of *FASN* and *ACACA* reflects increased transcription, we alternatively performed ChIP–qPCR and tested promoter histone acetylation of *LDHA* and *VEGF* (Supplementary Fig. 2g, h, i, j). The acetylation levels (H3K9ac, H3K27ac and H3K56ac) at *LDHA* and *VEGF* promoters were increased under hypoxia, which was further upregulated by acetate supplementation (Supplementary Fig. 2g, h, i, j). Consistently, mRNA expression of *LDHA* and *VEGF* was also activated by acetate (Supplementary Fig. 2k,l). These observations suggest that acetate contributes to histone acetylation at the promoters of hypoxia-induced genes. Collectively, these data support our notion that acetate promotes lipid synthesis pathway through epigenetic regulation.

### Acetate-induced lipogenesis does not reflect lipid demands

**Figure 3:**
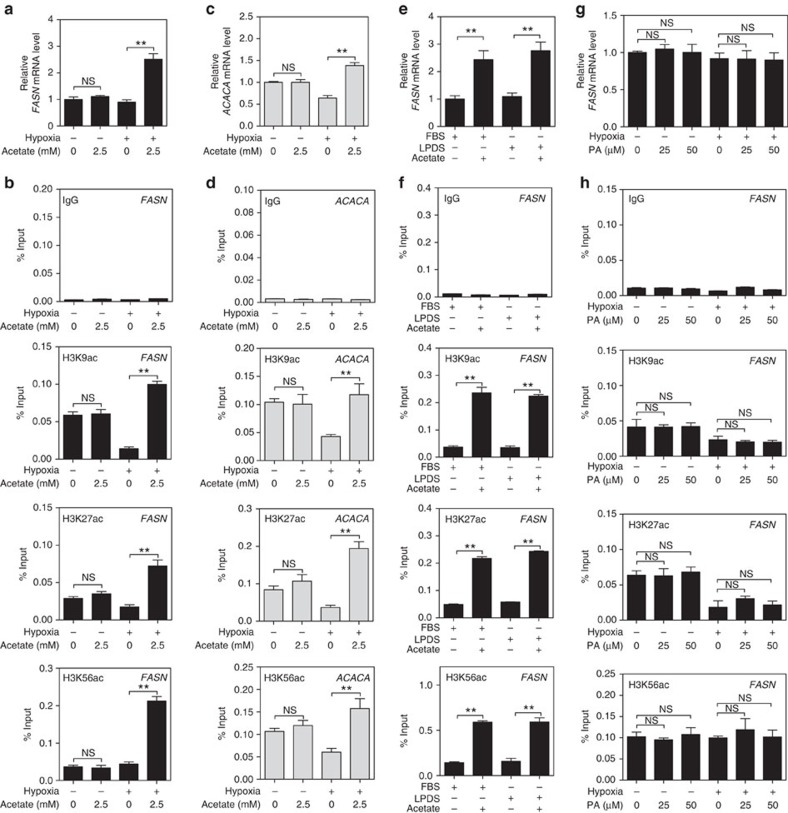
Acetate epigenetically activates lipogenic genes without reflecting cellular lipid demands. (**a**) *FASN* expression in HepG2 cells treated with indicated concentrations of acetate for 12 h under normoxia or hypoxia were quantified by qPCR. The results were presented as mean±s.d. of triplicate experiments (^**^P<0.01; NS, not significant; by Student's *t*-test). (**b**) ChIP-qPCR assays showing histone acetylation enrichment at *FASN* promoter region in HepG2 cells treated with indicated concentrations of acetate under normoxia or hypoxia for 4 h. Rabbit IgG was included as a negative control. Each histogram was presented as mean±s.d. of triplicate experiments (^**^P<0.01; NS, not significant; by Student's *t*-test). (**c**) *ACACA* expression in HepG2 cells treated as in panel (**a**) were quantified by qPCR. The results were presented as mean±s.d. of triplicate experiments (^**^P<0.01; NS, not significant; by Student's *t*-test). (**d**) ChIP-qPCR assays showing histone acetylation enrichment at *ACACA* promoter region in HepG2 cells treated as in **b**. Each histogram was presented as mean±s.d. of triplicate experiments (^**^P<0.01; NS, not significant; by Student's *t*-test). (**e**) Quantification of *FASN* expression in HepG2 cells treated with or without 2.5 mM acetate in media plus 10% FBS or 10% LPDS for 12 h under hypoxia by qPCR. The results were presented as mean±s.d. of triplicate experiments (^**^P<0.01; by Student's *t*-test). (**f**) ChIP-qPCR assays showing histone acetylation levels at *FASN* promoter region in HepG2 cells treated as in **e** for 4 h under hypoxia. The results were presented as mean±s.d. of triplicate experiments (^**^P<0.01; by Student's *t*-test). (**g**) Quantification of *FASN* expression in HepG2 cells treated with 10% LPDS plus the indicated concentration of palmitate (PA) for 12 h under normoxia or hypoxia by qPCR. The results were presented as mean±s.d. of triplicate experiments (NS, not significant; by Student's *t*-test). (**h**) ChIP-qPCR assays showing histone acetylation levels enrichment at *FASN* promoter region in HepG2 cells treated as in **g** for 4 h under normoxia or hypoxia. The results were presented as mean±s.d. of triplicate experiments (NS, not significant; by Student's *t*-test).

Cancer cells show increased demands for lipids, through scavenging extracellular lipids or *de novo* lipid synthesis^2^,^30^,^31^. We tried to figure out whether acetate-induced expression of *FASN* or *ACACA* is affected by the extracellular lipids under hypoxia. Consistent with data in Fig. 2, *FASN* and *ACACA* mRNA expression was more prone to be induced by acetate under hypoxia, compared with normoxia (Fig. 3a,c) and acetate-activated promoter histone acetylation of *FASN* and *ACACA* more pronouncedly under hypoxia (Fig. 3b,d). Furthermore, lipid depletion in the serum did not alter acetate-induced expression of *FASN* and *ACACA* in HepG2 cells (Fig. 3e; Supplementary Fig. 3a). Similarly, *FASN* and *ACACA* promoter histone acetylation was not affected by lipid depletion as well (Fig. 3f; Supplementary Fig. 3b). Moreover, palmitate supplementation did not change the expression of *FASN, VEGF* and *ACACA* (Fig. 3g; Supplementary Fig. 3c,d). Consistently, histone acetylation at the promoter regions of *FASN* and *ACACA* did not respond to palmitate supplementation (Fig. 3h; Supplementary Fig. 3e). These results demonstrate that acetate increases histone acetylation at promoters of lipogenic genes and activates their mRNA expression, without reflecting lipid demand of hypoxic cells.

### ACSS1 and ACSS2 are both involved in acetate-induced lipogenesis

**Figure 4:**
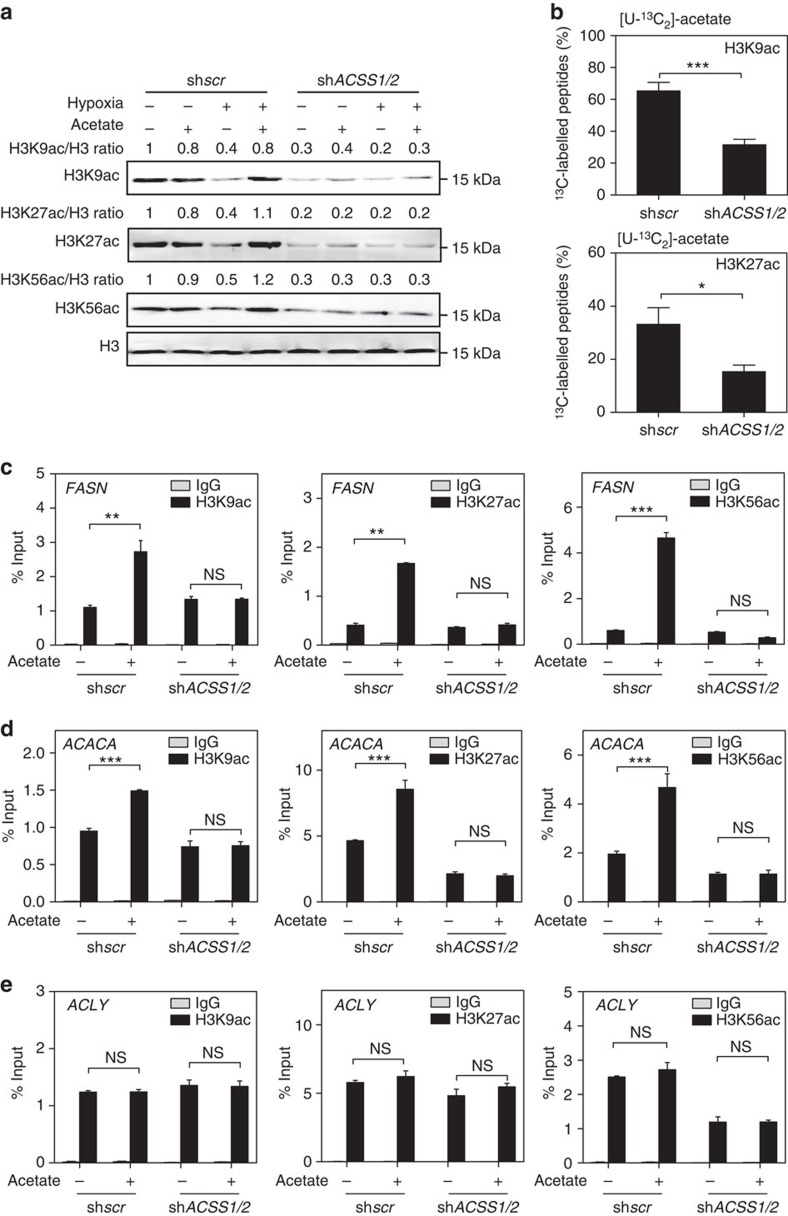
Both ACSS1 and ACSS2 are involved in acetate-induced epigenetic regulation of *de novo* lipogenesis. (**a**) Stably concurrent knockdown of *ACSS1* and *ACSS2* totally blocks acetate-induced increase of histone acetylation levels. Histone acetylation levels in sh*scr* or sh*ACSS1/2* HepG2 cells treated with or without 2.5 mM acetate under normoxia or hypoxia were analysed by western blot. (**b**) ^13^C_2_-labelled H3K9ac and H3K27ac levels are decreased by *ACSS1/2* knockdown. Quantification of ^13^C_2_-labelled histone H3 acetylation levels in sh*scr* or sh*ACSS1/2* HepG2 stable cell line treated with [U-^13^C_2_]-acetate for 4 h under hypoxia was analysed by LC-MRM MS. The percentage indicates the ratio of H3K[^13^C_2_-ac]/total H3Kac in each site. The results were presented as mean±s.d. of triplicate experiments (*P<0.05; ^***^P<0.001; by Student's *t*-test). (**c**,**d**) *ACSS1/2* knockdown totally blocks acetate-induced histone acetylation association with *FASN* and *ACACA* promoter. ChIP-qPCR assays were performed to determine H3K9, H3K27 and H3K56 acetylation enrichment at *FASN* (**c**) and *ACACA* (**d**) promoter region in sh*scr* or sh*ACSS1/2* HepG2 stable cell lines treated with or without acetate under hypoxia for 4 h. IgG was included as a negative control. The results were presented as mean±s.d. of triplicate experiments (^**^P<0.01; ^***^P<0.001; NS, not significant; by Student's *t*-test). (**e**) ChIP-qPCR assays were performed to determine H3K9, H3K27 and H3K56 acetylation enrichment at *ACLY* promoter region in sh*scr* or sh*ACSS1/2* HepG2 cells treated as in **c**. IgG was included as a negative control. The results were presented as mean±s.d. of triplicate experiments (NS, not significant; by Student's *t*-test).

In mammals, two main enzymes involve in acetyl-CoA production from acetate: the cytosolic ACSS2 and its mitochondrial paralogue ACSS1. To dissect which enzyme is responsible for mediating the acetate-induced increase of histone acetylation, we knocked down *ACSS1* or *ACSS2* individually and assessed the corresponding histone acetylation levels. To our surprise, knockdown of either *ACSS1* or *ACSS2* was incapable of blocking acetate-induced increase in acetylation levels at H3K9, H3K27 and H3K56 sites (Supplementary Fig. 4a,b). Though discrepant with the other studies reporting that ACSS2, but not ACSS1, appeared to be the key enzyme involved in metabolizing acetate for lipid synthesis^5^,^9^, our findings were consistent with those by Yun *et al.*^32^ and Bjornson *et al.*^33^, which reported that ACSS1 was also vital for acetate-dependent lipid synthesis. These results indicate that ACSS1 and ACSS2 are functionally redundant. We then established *ACSS1*/*2* double knockdown stable cell pools. Concurrent knockdown of *ACSS1*/*2* by using two different sets of shRNAs dramatically inhibited acetate-induced increase in acetylation levels of H3K9, H3K27 and H3K56 under hypoxia (Fig. 4a; Supplementary Fig. 4c). Moreover, we conducted quantification based on LC-MRM MS to see whether *ACSS1*/*2* double knockdown could reduce the abundance of histone acetylation peptides derived from acetate by using [U-^13^C_2_]-acetate tracer. Acetate-derived acetyl-CoA was found actively incorporated into H3K9, H3K14 and H3K27 acetylation sites, compared with H3K18, H3K23 and H3K56 acetylation sites (Fig. 4b; Supplementary Fig. 4d). These results demonstrate that acetate could function as an acetyl donor to induce histone acetylation. *ACSS1*/*2* double knockdown induced a more significant decrease in ^13^C_2_-labelled acetylated H3K9, H3K27 peptides derived from [U-^13^C_2_]-acetate (Fig. 4b; Supplementary Fig. 4d). As for the discrepancy of H3K56 acetylation between western blot data and MS quantification, it might be due to the fact that concentration of ^13^C_2_-labelled acetylated H3K56 peptide was too low to meet the limit of quantification. In addition, we designed experiments to test whether *ACSS1*/*2* double knockdown could block acetate-induced histone acetylation at *FASN* and *ACACA* promoter regions. *ACSS1/2* knockdown efficiency was verified by qPCR (Supplementary Fig. 4e). We found that acetate-increased H3K9, H3K27 and H3K56 acetylation levels at *FASN* (Fig. 4c) and *ACACA* (Fig. 4d) promoter regions were blocked by *ACSS1*/*2* double knockdown under hypoxia. *ACSS1*/*2* knockdown had no effect on histone acetylation levels at *ACLY* promoter region (Fig. 4e). Consistently, *ACSS1/2* knockdown blocked acetate-induced *FASN* expression (Supplementary Fig. 4f). Collectively, our data demonstrate that acetate contributes to histone acetylation in an ACSS1/2-dependent manner under hypoxia.

Previous study linked ACLY-dependent glucose metabolism to histone acetylation^1^,^22^. To dissect contributions of different carbon sources to histone acetylation, we performed qPCR and ChIP–qPCR assays in *ACSS1/2*-knockdown and/or *ACLY*-knockdown cells. The knockdown efficiency was verified (Supplementary Fig. 4g). Depletion of either *ACSS1/2* or *ACLY* reduced gene expression of *LDHA* and *VEGF*, which were further decreased by knockdown of both *ACSS1/2* and *ACLY* (Supplementary Fig. 4h,i). Consistently, either *ACSS1/2* or *ACLY* knockdown reduced histone acetylation at the promoter regions of *LDHA* and *VEGF* (Supplementary Fig. 4j, k, l, m). These results demonstrate that both ACSS1/2 and ACLY contribute to histone acetylation under hypoxia.

### Acetate-induced lipogenesis promotes cell survival

**Figure 5:**
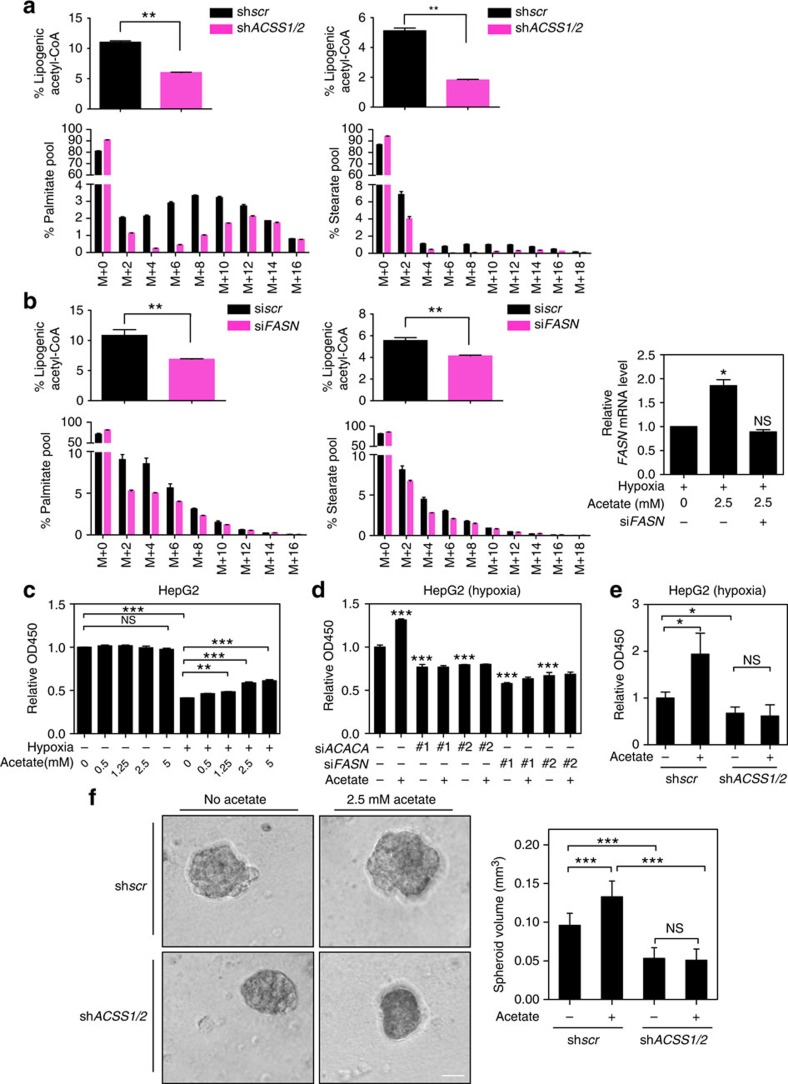
Epigenetic regulation of *de novo* lipogenesis by acetate is vital for cell survival under hypoxia. (**a**) ACSS1/2 mediates acetate-induced palmitate and stearate production. Fractional abundance of palmitate (left) and stearate (right) isotopologues in *ACSS1/2* stably double-knockdown HepG2 cells (designated as magenta) compared with that of control cells (designated as black) after 24 h of culture under hypoxia with [U-^13^C_2_]-acetate. Each histogram was presented as mean±s.d. of triplicate experiments. The inset shows the percentage of lipogenic acetyl-CoA derived from acetate carbon (^**^P<0.01; by Student's *t*-test). (**b**) Fractional abundance of palmitate (left panel) and stearate (middle panel) isotopologues in scramble (si*scr*) and si*FASN* HepG2 cells treat as in **a**. Each histogram was presented as mean±s.d. of triplicate experiments. The inset shows the percentage of lipogenic acetyl-CoA derived from acetate carbon. The knockdown efficiency was detected by PCR with reverse transcription (right panel) (^**^P<0.01; NS, not significant; by Student's *t*-test). (**c**)Acetate promotes cell survival under hypoxia. Viability of HepG2 cells treated with indicated concentrations of acetate under normoxia or hypoxia was determined via CCK8 assay. The results were presented as mean±s.d. of triplicate experiments (^**^P<0.01; ^***^P<0.001; NS, not significant; by Student's *t*-test). (**d**) Viability of control or si*FSAN* or si*ACACA* HepG2 cells treated with or without 2.5 mM acetate under hypoxia was determined via CCK8 assay. The results were presented as mean±s.d. of triplicate experiments (^***^P<0.001; by Student's *t*-test). (**e**) ACSS1/2 mediates the promotion of cell survival by acetate. Viability of sh*scr* or sh*ACSS1/2* HepG2 stable cell lines treated as indicated was determined via CCK8 assay. The results were presented as mean±s.d. of triplicate experiments (*P<0.05; NS, not significant; by Student's *t*-test). (**f**) ACSS1/2 mediates cells growth advantage by acetate in Matrigel. Sh*scr* or sh*ACSS1/2* HepG2 cells were plated into the Matrigel bed and cultured with DMEM with 10% FBS, and 2% Matrigel for 12 days before the images were taken (left). Scale bar, 0.25 mm. The quantitative data were presented as mean±s.d. (*n*>3) (right) (^***^P<0.001; NS, not significant; by Student's *t*-test).

To test whether acetate would affect lipid pool in cancer cells, we cultured HepG2 stable cells in media with isotope-labelled [U-^13^C_2_]-acetate for 24 h under hypoxia and measured the isotopologues of fatty acids pool by using gas chromatography coupled with mass spectrometry (GC-MS). *ACSS1/2* double knockdown indeed decreased the fractional contribution of acetate to palmitate and stearate synthesis (Fig. 5a). These results are consistent with the model that acetyl-CoA derived from acetate stimulates *de novo* lipid synthesis^5^,^7^,^9^. Furthermore, we knocked down *FASN* expression to the unstimulated state by using the low-efficiency siRNA as in Supplementary Fig. 5a, and treated cells with isotope-labelled [U-^13^C_2_]-acetate under hypoxia. The *de novo* lipid synthesis from acetate-derived carbon was reduced in *FASN* knockdown cells, demonstrating that acetate functions as an acetyl donor to promote lipogenesis and acetate-induced *FASN* expression plays an important role in lipogenesis (Fig. 5b). Given that elevated lipid synthesis could promote tumour cell survival under hypoxia^2^,^25^, we carried out CCK8 assay and found that acetate supplement enhanced cell survival under hypoxia, but not normoxia (Fig. 5c). To interrogate whether acetate promoted cancer cell survival through regulating lipogenic genes, we employed specific siRNAs targeting *FASN* or *ACACA*, of which the relative low knockdown efficiencies are capable of suppressing acetate-simulated *FASN* and *ACACA* mRNA expression to the level similar to unstimulated state (Supplementary Fig. 5a). We found that acetate-induced cell survival under hypoxia was inhibited by knockdown of either *FASN* or *ACACA*, indicating that acetate-promoted cell survival was dependent on lipogenesis and the induction of lipogenic gene expression was vital for cell survival (Fig. 5d). Strikingly, we found that inhibition of either *FASN* or *ACACA* to the untreated level had no effect on acetate-induced *FASN* promoter histone acetylation (Supplementary Fig. 5b). We attempted to further dissect whether acetate-induced *de novo* lipid synthesis is vital for cancer cell cultured under hypoxia. We found that HepG2 and SkBr3 cells were more sensitive to C75, a chemical inhibitor of FASN, when cultured under hypoxia (Supplementary Fig. 5c), consistent with previous report that cancer cells were dependent on *de novo* lipid synthesis under hypoxia^9^. Moreover, C75 treatment abrogated acetate-induced cancer cell survival without affecting *FASN* and *ACACA* mRNA levels (Supplementary Fig. 5d,e,f). Consistently, *ACSS1/2* knockdown blocked the increased cell survival induced by acetate supplement, indicating that acetate-induced cell survival was *ACSS1/2*-dependent (Fig. 5e). In addition, we performed Matrigel assay and found that HepG2 cells supplied with acetate exhibited growth advantage over the control group, and this effect was blocked by *ACSS1/2* double knockdown (Fig. 5f). Taken together, beyond functioning as a precursor for lipogenesis, acetate also serves as acetyl donor to induce histone acetylation and mRNA expression of lipogenic genes, contributing to the adaptation of cancer cells to hypoxia.

### ACSS1/2 positively correlates with FASN expression in HCC

**Figure 6:**
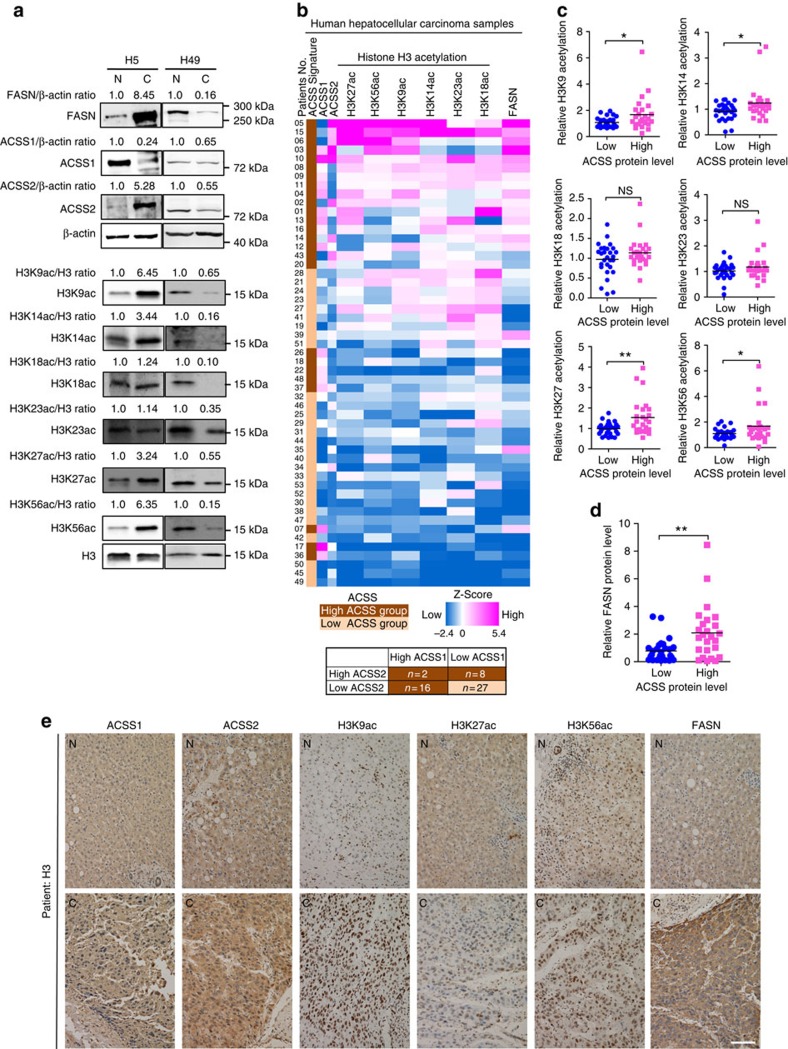
ACSS1/2 positively correlates with histone acetylation and FASN expression in human hepatocellular carcinoma. (**a**) FASN, ACSS1 and ACSS2 protein levels (upper panels, normalized against β-actin) and H3K9, H3K14, H3K18, H3K23, H3K27 and H3K56 acetylation levels (lower panels, normalized against histone H3) in 53 pairs of HCC (each paired with cancerous tissue (designated as C) and adjacent normal tissue (designated as N)) were analysed by western blot. Two pairs of representative samples were shown. For the other 51 pairs of samples, please refer to Supplementary Fig. 6b. (**b**) Heatmap of protein expression (ACSS1, ACSS2 and FASN) and histone acetylation levels (H3K9ac, H3K14ac, H3K18ac, H3K23ac, H3K27ac and H3K56ac) in all 53 pairs of HCC. Data were presented as *Z*-score of relative protein expression or histone acetylation. Tumours with 1.5-fold higher expression of ACSS1 or ACSS2 or both than that of adjacent normal control tissue are grouped into ACSS-high tumours (tumour/normal ≥1.5, *n*=26), while ACSS-low tumours (tumor/normal<1.5, *n*=27) express both ACSS proteins at 1.5-fold lower than its normal control. (**c**) H3K9ac (*P*=0.0220), H3K14ac (*P*=0.0289), H3K27ac (*P*=0.0034), and H3K56ac (*P*=0.0410), are significantly stronger in ACSS-high tumours than that in ACSS-low tumours. Statistical analyses were performed with a two-tailed unpaired Student's *t*-test (*P<0.05; ^**^P<0.01; NS, not significant). (**d**) FASN protein level is significantly upregulated in ACSS-high tumours (*P*=0.002). Statistical analyses were performed with a two-tailed unpaired Student's *t*-test (^**^P<0.01). (**e**) Representative immunohistochemical staining results for ACSS1, ACSS2, FASN and H3K9/K27/K56 acetylation in adjacent normal tissue (N) and hepatocellular carcinoma (C). Scale bar, 100 μm.

ACSS2 is reported to be upregulated in various human cancers^3^,^5^,^9^. ACSS1 is also reported to be increased in hepatocellular carcinoma^33^. Through an analysis of 190 human HCC samples data from the Cancer Genome Atlas (TCGA)^34^, we found a significant correlation between *FASN* and *ACSS2* mRNA levels and no significant correlation between *FASN* and *ACSS1* mRNA levels (Supplementary Fig. 6a). Our aforementioned results demonstrated that acetate could epigenetically regulate *FASN* expression, which prompts us to examine ACSS1/2, FASN, and H3 acetylation levels and their correlation in human HCC. We collected 53 pairs of primary human HCC samples with adjacent normal tissues, and determined protein changes (ACSS1, ACSS2 and FASN) and six histone acetylation marks (H3K9ac, H3K14ac, H3K18ac, H3K23ac, H3K27ac and H3K56ac) in all these samples (*n*=53) (Fig. 6a; Supplementary Fig. 6b). To perform a statistical analysis of the association between ACSS1/2 and histone acetylation, we divided tumour samples into two groups based on ACSS1/2 signature (high versus low). Tumours with 1.5-fold higher expression of ACSS1 or ACSS2 or both than that of adjacent normal control tissue are grouped into ACSS high-expression tumours (tumour/normal ≥1.5, *n*=26), while ACSS low-expression tumours (tumour/normal <1.5, *n*=27) express both ACSS proteins at 1.5-fold lower than its normal control (Fig. 6b). We found 16 cases with ACSS1 high expression, 8 cases with ACSS2 high expression and 2 cases with both ACSS1 and ACSS2 high expression (Fig. 6b). The higher percentage of ACSS1 high-expression cases than that of ACSS2 fully proves the importance of ACSS1 in acetate utilization by cancer. Our results demonstrate that four out of six histone acetylation marks, including H3K9ac (*P*=0.0220), H3K14ac (*P*=0.0289), H3K27ac (*P*=0.0034) and H3K56ac (*P*=0.0410), are significantly stronger in ACSS high-expression tumours than that from ACSS low-expression tumours with two-tailed unpaired Student's *t*-test (Fig. 6c). In line with this observation, FASN protein level is also significantly upregulated in ACSS high-expression tumours (Fig. 6d). Consistently, we performed immunohistochemistry (IHC) analysis and found that ACSS1/2 expression was positively correlated with H3 acetylation and FASN expression in human HCC. A representative case was shown in Fig. 6e. By using Spearman correlation analysis, we have evaluated the relationship between histone acetylation and FASN expression. Surprisingly, each and every histone acetylation mark in our analysis exhibits significant positive correlation with FASN expression (*P*<0.0001 for H3K9ac, *P*=0.0003 for H3K14ac, *P*=0.0009 for H3K18ac, *P*=0.0385 for H3K23ac, *P*<0.0001 for H3K27ac and *P*<0.0001 for H3K56ac; Supplementary Fig. 6c). Collectively, these results strongly support a positive correlation between ACSS1/2, FASN and histone acetylation in human HCC.

## Discussion

Acetyl-CoA is a central metabolite derived mainly from glucose, glutamine and fatty acids in mammals. However, the capability of cancer cells to produce acetyl-CoA from these conventional carbon sources is dramatically decreased under hypoxia^3^,^7^. Cancer cells, therefore, need alternative carbon sources and conduct metabolic reprogramming. Clinical studies report the increase in [^11^C]-acetate uptake in multiple types of cancers including prostate, liver, lung and brain tumours. Recently, new studies further demonstrate that various tumours consume exogenous acetate to generate acetyl-CoA for lipid synthesis^3^,^7^,^9^. These studies support that acetate serves as an important carbon source for cancer cells under unfavourable conditions. The underlying mechanism of how acetate functions as carbon source to fuel tumour growth, however, remains as an open question.

**Figure 7:**
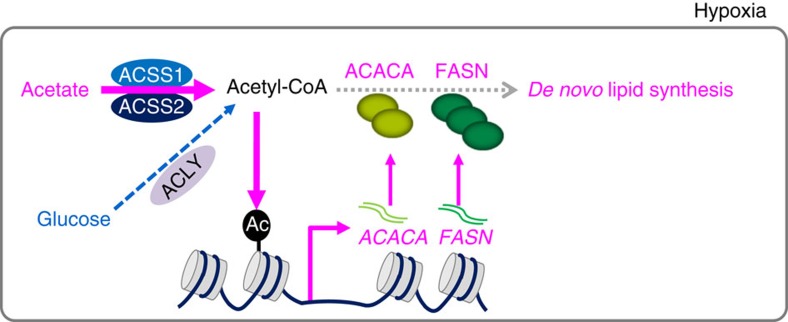
The model for acetate-induced epigenetic regulation of *de novo* lipogenesis. In addition to its ability to induce fatty acid synthesis as an immediate metabolic precursor, acetate also functions as an epigenetic metabolite to induce H3 acetylations in both dose- and time-dependent manners under hypoxia, enhancing H3K9, H3K27 and H3K56 acetylation levels at *FASN* and *ACACA* promoter regions, which upregulates *FASN* and *ACACA* expression and increases *de novo* lipid synthesis to promote tumour cell survival.

In this study, we elucidate that acetate functions as an epigenetic regulating metabolite to enhance lipid synthesis and to promote tumour survival under hypoxia. First, histone acetylation at H3K9, H3K27 and H3K56 sites can be stimulated by acetate in both time- and dose-dependent manners under hypoxia in mutiple cancer cell lines. Notably, acetate-derived acetyl-CoA is found to be incorporated not only into fatty acid synthesis but also into acetylated histone peptides. Second, acetate-induced histone acetylation is then associated with *FASN* and *ACACA* promoter regions, which activates *FASN* and *ACACA* expressions under hypoxia. Third, acetate-driven epigenetic regulation is mediated by ACSS1 and ACSS2. Last and most important, acetate-induced lipogenic genes expression is found to promote *de novo* lipid synthesis and cell survival under hypoxic stress. Collectively, besides its ability to induce fatty acid synthesis as an immediate metabolic precursor, our study reveals a new mechanism of epigenetic regulation by acetate to increase lipid synthesis and promote cell survival under unfavourable conditions (Fig. 7). However, we assess effects of acetate using limited set of cell lines *in vitro*, it needs further confirmation of the findings using more cell lines. In addition, the experiments *in vivo* would provide more validation.

Alterations in histone modifications play important roles in transcriptional regulation in cancer cells^35^. H3K9ac, H3K27ac and H3K56ac, three epigenetic marks of active gene transcription, are found to be altered in various cancers. H3K9 hyperacetylation is associated with specific genes in breast cancer^36^. Similarly, H3K27 acetylation is upregulated in colorectal cancer^37^. Intensive investigations also show that H3K56 acetylation is closely related with epigenetic activation and is increased in multiple types of cancer^38^,^39^,^40^,^41^,^42^,^43^. In our work, we found that H3K9, H3K27 and H3K56 acetylation levels were increased in ACSS1/2-overexpressed human HCC, showing a significantly positive correlation between ACSS1/2 expression, histone acetylation and FASN expression. ACSS1/2 upregulation suggests that these cancer cells utilize acetate as both epigenetic metabolite and carbon source to meet their growth needs. These findings validate our model *in vivo*: ACSS1/2 links histone acetylation with FASN expression and acetate-induced epigenetic regulation may play a pivotal role in human HCC. As the major enzymes in acetate metabolism, ACSS1 and ACSS2 may merit further explorations as a therapeutic target for cancer.

Dysregulation of *de novo* lipid synthesis is a hallmark of cancer^44^. Increased lipid synthesis fuels cancer engine and contributes to cancer cells survival when facing with stressed conditions, such as hypoxia^25^,^30^,^44^,^45^,^46^,^47^,^48^,^49^. Upregulation of FASN is common in many cancers and FASN is targeted for cancer therapy^44^,^49^,^50^,^51^. FASN inhibition with chemical inhibitors or RNAi can suppress tumour cell survival^51^. FASN upregulation in cancers is in part due to the transcriptional activation by SREBPs^44^,^45^,^52^,^53^. In addition, USP2a was reported to enhance FASN protein stabilization^44^. Our study proves that acetate increases histone acetylation at promoters of lipogenic genes and activates their mRNA expression, without reflecting lipid demand of hypoxic cells. Interestingly, our data show that both ACSS1/2-mediated acetate and ACLY-mediated glucose contribute to histone acetylation at the promoters of hypoxia-induced genes. *VEGF* or *LDHA* shows a similar response that occurs in hypoxia alone without acetate addition yet each remains responsive to acetate. This suggests the changes occurring at these promoters that lead to gene activation (presumably recruitment of co-activators) render them more sensitive to acetyl-CoA levels compared with other genes. How recruitment of co-activators modulates acetate-induced lipogenic genes expression is an interesting question.

In conclusion, our study adds a novel mechanism of epigenetic regulation to *FASN* upregulation. Besides glucose, cancer cells absorb more acetate under hypoxia. Besides functioning as an immediate metabolic precursor, acetate-derived acetyl-CoA increases H3K9, H3K27 and H3K56 acetylation levels at *FASN* and *ACACA* promoter regions, initiating epigenetic regulation of *FASN* and *ACACA*. Due to the limitation of our system in distinguishing the role of acetyl-CoA as a substrate rather than an epigenetic regulator, both pathways cannot be separated well and their relative contribution remains unclear. Histone acetylation modulated by acetate status might play a pivotal role in coordinating acetate and glucose availability with the intracellular level of *FASN*, hence fatty acid synthesis. Taken together, this study demonstrates that acetate, in addition to its ability to induce fatty acid synthesis as an immediate metabolic precursor, also functions as an epigenetic metabolite to promote cancer cell survival under hypoxic stress.

## Methods

### Cell culture

PLC-8024 cells were obtained from the Institute of Virology, Chinese Academy of Medical Sciences (Beijing, China). All the other cell lines were obtained from the Cell Bank of Type Culture Collection of Chinese Academy. HepG2, SkBr3, PLC-8024 and DU 145 cells were grown in DMEM medium (Gibco, high glucose) with 10% fetal bovine serum (FBS, biological industries (BI)), and penicillin/streptomycin. A549 cells were grown in F12K medium (Sigma) with 10% FBS (BI), and penicillin/streptomycin. HT29 cells were cultured in McCoy's 5A medium (Sigma) with 10% FBS (BI), and penicillin/streptomycin. Mycoplasma contamination check was carried out on cultured cells. Hypoxia condition (1% O_2_, 94% N_2_, 5% CO_2_) was achieved by gas chamber (C42, Biospherix).

### Antibodies and reagents

Anti-histone H3 (9715S, CST), anti-H3K9 acetyl (1328-1, Epitomics), anti-H3K27 acetyl (EP865Y, Epitomics), anti-H3K56 acetyl (EPR996Y, Epitomics), anti-ACSS2 (ab133664, Abcam), anti-ACSS1 antibody (17138-1-AP, Proteintech), anti-FASN (C-20140, Santa Cruz), anti-ACLY antibody (1699-1, Epitomics) and anti-β-actin (a00702, Genescript) antibodies were commercially obtained. ChIP grade antibodies for H3K9 acetyl (ab4441, Abcam), H3K27 acetyl (ab4729, Abcam), H3K56 acetyl (17-10259, Milipore) were commercially obtained.

[U-^13^C_2_]-Acetate (CLM-440-1, Cambridge Isotope Laboratories), lipoprotein depleted fetal bovine serum (LPDS) (880100, Kalen Biomedical), sodium palmitate (P9767, Sigma), BSA with low fatty acid (0219989925, MP) and TSA (9950, CST) were commercially obtained. Myristic acid (D27, 98%) as internal standard was obtained from Cambridge Isotope Laboratories (Tewksbury, MA, USA). Certified ACS grade sodium hydroxide was purchased from Standard Chemical and hydrochloric acid was from VWR International LLC (Pennsylvania, USA). Formic acid and BSTFA (with 1% TMCS) were purchased from Sigma-Aldrich. Methanol and chloroform were obtained from Tedia (Fairfield, USA) and hexane obtained from Duksan Pure Chemicals Co., Ltd (South Korea).

### Western blotting

Standard western blot protocols were adopted. The quantification was carried out by subtracting background from the band intensity of western blots by using software ImageQuantTL (GE).

### LC-MRM MS quantification of histone acetylation

Histone proteins were extracted and purified as previously described by Gao *et al*^54^. Briefly, 3 × 10^6^ scramble (sh*scr*) or sh*ACSS1/2* HepG2 cells were cultured in DMEM medium (without glucose or glutamine, Sigma) by adding 10% dialysed FBS (BI), 2.5 mM [U-^13^C_2_]-acetate, 5 mM label-free glucose, 2 mM label-free glutamine for 4 h under hypoxia (1% O_2_), followed by lysis with hypotonic lysis buffer (10 mM Tris-HCl (pH 8.0), 1 mM KCl, 1.5 mM MgCl_2_, 1 mM DTT and protease inhibitors) to isolate nuclei. Crude histone was extracted with 0.4 N HCl overnight. After removal of HCl by ultrafiltration, the crude histone was processed to derivatization in a final condition of 100 mM NHS-propionylate ester (home-made), 25 mM NH_4_HCO_3_, 50% ACN, 50 °C for 30 min. The proteins were SpeedVac to dryness and digested with trypsin in 25 mM NH_4_HCO_3_ overnight. Finally, the digested peptides were concentrated to dryness again and derivatized for a second round of histone NHS propionylation.

Stable isotope-labelled histone peptides containing various PTMs and chemical derivatization (purity >95%) were from New England Peptide LLC (Gardner, MA, USA). Prior to LC-MS analysis, internal standards were mixed with the diluted derivatized histone samples in a ratio of 1:2 in volume. In the final solution, the internal standard was doped as 25 nM stable isotope-labelled histone peptides, while the final concentration of stable isotope-labelled peptides with K27 acetylation was 2.5 nM. The peptides were first trapped to a nanoLC trap column at 2 ml min^−1^ liquid flow that was diverted from the analytical column via a vent valve, while separation was performed by switching the valve to make the trap column in line with a C18 analytical column with a flow rate of 300 nl min^−1^. The chromatographic gradient was 5–35% B from 0 to 45 min, 35–80% B from 45 to 60 min, and maintaining 80% B for 4 mins (mobile phase A: 0.1% formic acid in ddH_2_O; mobile phase B: 0.1% formic acid in acetonitrile). Then, the peptides were directly analysed online using QTRAP 6500 mass spectrometer (Applied Biosystems, Singapore) set to multiple reaction monitoring (MRM) in positive electrospray ionization mode. The mass transitions were selected as previously described by Chen *et al*^54^,^55^, with the addition of transitions corresponding to ^13^C_2_-labelled acetylated peptides. All MRM data were processed through Skyline software (Washington University in St Louis, St Louis, MO, USA). The quantification was based on the peak area and the measured areas under the curve of indicated product ions were calculated. All data were manually inspected to ensure correct peak detection and accurate integration according to four criteria: (1) the correct *m/z* is selected for both the heavy and light traces of each peptide, and [U-^13^C_2_]-acetylation for acetylated peptides; (2) the peak shapes are Gaussian-like and do not show excessively jagged appearance; (3) the retention time of certain peptide is similar between different runs; and (4) the relative contribution of each transition to the total signal is similar between each sample.

### RNA extraction and qPCR

Total RNA was extracted with Trizol extraction kit (Invitrogen). qPCR was performed using SYBR Premix Ex Taq (TakaRa) in an ABI 7500 Sequence Detection System (Applied Biosystems). All reactions were performed in triplicate and relative mRNA expression was normalized to β-actin. All tested primers are listed in Supplementary Table 1.

### ChIP–qPCR assays

ChIP–qPCR assays were performed as previously described^56^. Briefly, 1 × 10^6^ HepG2 cells were cross-linked with 1% paraformaldehyde, lysed and sonicated using the Bioruptor at high-output power setting for eight cycles (30 s ON and 30 s OFF). Solubilized chromatin was immunoprecipitated with chip grade antibodies for H3K9 acetyl, H3K27 acetyl, H3K56 acetyl or rabbit IgG (negative control), following that the antibodies were preincubated with protein protein A sepharose overnight at 4 °C. Antibody–chromatin complexes were pulled down by protein A sepharose (Santa Cruz), washed and then eluted. After crosslink reversal in a water bath at 65 °C overnight and proteinase K treatment for 2 h at 55 °C, the immunoprecipitated DNA was extracted with phenol–chloroform, and ethanol precipitated. The DNA fragments were detected by qPCR. Histone acetylation marks were mapped at promoters spanning −3 to 3 kb of target genes (*ACACA*, *FASN*, *ACLY*, *VEGF* and *LDHA*). Primers spanning the regions with peaks were adopted for ChIP–qPCR analysis. All tested primers targeting *FASN*, *ACACA*, *ACLY*, *LDHA* and *VEGF* are listed in Supplementary Table 1.

### RNA interference

For RNA interference experiments, si *ACSS1* #1 (EHU040311) and si *ACSS2* #1 (EHU135341) were purchased from Sigma. The others were obtained from commercial synthetic siRNA oligonucleotide (Genepharma, Shanghai).

si*ACSS1* #2: 5′-UCACCGUAUUUCAGCAACAGCCGG-3′;

si*ACSS2* #2: 5′-UAUGCUUGGUGACAGGCUCAUCUCC-3′;

si*ACLY* #1: 5′-GAUCAAACGUCGUGGAAAAUU-3′;

si*ACLY* #2: 5′-GAGGAAGCUGAUGAAUAU-3′;

si*FASN* #1: 5′-UGGAGCGUAUCUGUGAGAAUU-3′;

si*FASN* #2: 5′-AACCCUGAGAUCCCAGCGCUG-3′;

si*ACACA* #1: 5′- CAAUGGCAUUGCAGCAGUGUU-3′;

si*ACACA* #2: 5′-UAUGAGGUGGAUCGGAGAUUUUU-3′.

All siRNA transfection experiments were performed with Lipofectamine RNAiMAX Transfection Reagent (Invitrogen) according to the reverse transfection instructions. A volume of 5 μl siRNA (10 μM) plus with 5 μl Lipofectamine RNAiMAX per well (six wells) was used. The cell density was 8 × 10^5^ HepG2 cells per well (six wells). The fresh culture media was exchanged after 24 h. The samples with indicated treatment were collected at 72 h after transfection. The knockdown efficiency were detected by western blot or qPCR.

### Generation of stable knockdown cell lines

The retroviral knockdown constructs of sh*ACSS1* were constructed in pMKO.1-hyg vector as previously described^57^. The lentiviral knockdown constructs of sh*ACSS2* were constructed in pLKO.1-puro vector. The targeting sequences were as follows:

sh*ACSS1*#1: 5′-TCACCGTATTTCAGCAACAGCCGG-3′;

sh*ACSS1*#2: 5′-AGGTGGTTATCACCTTCAA-3′;

sh*ACSS2*#1: 5′-TACAATGTACTGGATCGAA-3′;

sh*ACSS2*#2: 5′-AACGCTTTGAGACAACCTA-3′;

The virus was packaged in HEK293T cells by co-transfecting pLKO.1-sh*ACSS2* or pLKO.1-sh*ACSS2* or corresponding vectors (pLKO.1-sh*scr* and pLKO.1-sh*scr*) with package vectors expressing gag and vsvg genes (from vesicular stomatitis virus G). The virus in the culture media was harvested every 24 h for 48 h after initial plasmid transfection. For infection, HepG2 cells were infected for 12 h with pMKO.1-sh*ACSS1* and pMKO-sh*scr* retrovirus with 8 μg ml^−1^ polybrene to increase the infection efficiency and selected in 200 μg ml^−1^ hygromycin B for 1 week first. Then the stable sh*ACSS1* or sh*scr* HepG2 cells were infected with pLKO.1-sh*ACSS2* and pLKO.1-sh*scr* retrovirus and selected in 1 μg ml^−1^ puromycin for 1 week to obtain *ACSS1* and *2* double knockdown (sh*ACSS1/2*) stable cell lines. The knockdown efficiency was verified by western blot.

### NMR spectroscopy

To detect acetate uptake in cancer cells, the cancer cells were grown in DMEM (Gibco, high glucose) supplemented with 10% FBS (BI) and penicillin/streptomycin. A volume of 200 μl of each medium was transferred into a 1.5-ml Eppendorf tube, then 600 μl of cold CD_3_OD with 0.09 mg ml^−1^ trimethylsilyl propionate (TSP) was added to the sample and vortexed for 30 s. The mixture was centrifuged at 20,817*g* for 10 min at 4 °C. Then 600 μl each supernatant was transferred to a standard 5-mm NMR tube for analysis.

To quantitatively analyse acetate in HCC tissues and adjacent normal tissue, 150 mg was weighted for each wet tissue. By adding 700 μl methanol and 280 μl H_2_O into each sample, homogenization was carried out by using TissueLyser (Qiagen, Germany) for 90 s at 20 Hz. Subsequently, more 700 μl chloroform and 350 μl H_2_O were added for further homogenization. The homogenate was centrifuged for 10 min at 10,621*g* at 4 °C. The supernatant was dried under gentle nitrogen stream. And then the dried residue was added with 600 μl D_2_O containing 0.05% TSP, vortexed and centrifuged for 8 min at 20,817*g*. The supernatant was removed into a standard 5-mm NMR tube for analysis.

One-dimensional NMR (^1^H-NMR) spectra were obtained on a 600-MHz Bruker Avance III HD spectrometer equipped with a 5-mm PABBO BB-probe. The spectra were acquired with presaturation of the water signal using the Bruker pulse program ‘noesygpprld' with a 16-p.p.m., 9,590.793 Hz width and 4.27 s acquisition time. All spectra were recorded with 81,920 data points at 25 °C. The time domain data were Fourier-transformed, phase-corrected and baseline-corrected manually. The signal intensities were normalized against the intensity of the TSP signal at 0.00 p.p.m.

### GC–MS analysis of lipid pool

HepG2 stable cells (5 × 10^6^) were cultured in DMEM medium (without glucose or glutamine, Sigma) by adding 10% dialysed FBS (BI), 25 mM label-free glucose, 4 mM label-free glutamine and 5 mM [U-^13^C_2_]-acetate for 24 h under 1% O_2_. Cells were rapidly washed twice with 37 °C PBS, then 1 ml PBS was added to the dish and quickly detached from the dish using a cell lifter. The liquid-containing cells were transferred into a 2-ml tube and centrifuged at 15,294*g* for 10 min at 4 °C. The supernatant was discarded and the residue was added to 400 μl of cold (80:20 methanol: water) extraction solution for quenching. The sample was vortexed and then stored at −20 °C for 1 h. The samples will be vortexed and centrifuged at 15,294*g* for 10 min at 4 °C. The supernatant will be moved to new tubes for dryness and used for further detection.

The sample preparation of the saponified fatty acids has been described previously^7^. Briefly, 1 ml MeOH/H_2_O (1:1, v/v) solution with 0.1 M HCl and 1.6 p.p.m. internal standard (myristic acid-D27) in solution was added into cell debris at 0 °C, vortexed for 30 s, followed by addition of 0.5 ml chloroform, and vortexed for 1 min, then centrifuged at 12,000*g* for 5 min at 4 °C. The resulting chloroform layer was transferred to a new glass vial, and the extract was dried under N_2_. The desiccation was reconstituted into 1 ml MeOH/H_2_O (9:1, v/v) containing 0.3 M NaOH, and incubated at 80 °C for 1 h to saponify the fatty acids. Then 0.1 ml of formic acid was added for acidification, followed by extraction twice with 1 ml hexane. The hexane layer was transferred to an Eppendorf tube for dryness under N_2_. The dried residue was added to 20 μl of pyridine and 80 μl of BSTFA (with 1% TMCS) and vortexed for 30 s, then derivatized at 70 °C for 1 h. The mixture was vortexed for 30 s and centrifuged at 12,000*g* for 5 min at room temperature before GC–MS analysis.

Analysis was performed using an Agilent 7890B gas chromatography system coupled to an Agilent 5977A mass spectrometric detector (MSD, Agilent Technologies). Derivatives were separated using an Agilent 122-5532 UI, DB-5MS capillary column (30 m × 250 μm × 0.25 μm). The injection volume was 1 μl in split mode in the ratio 5:1, and the solvent delay time was set to 6 min. The initial oven temperature was set at 100 °C for 1 min, then ramped to 200 °C at a rate of 20 °C min^−1^, to 260 °C at a rate of 10 °C min^−1^, then to 300 °C at a rate of 20 °C min^−1^ and finally set at 300 °C for 5 min. Helium was used as a carrier gas at a constant flow rate of 1 ml min^−1^ through the column. The temperatures of the front inlet, Aux-2 temperature and electron MS source were set at 280 °C, 250 °C and 230 °C, respectively. The electron energy was 70 eV, and the mass spectral data were collected in a full-scan mode (*m/z* 50–600). The mass isotopomer distribution of ^13^C-labelled palmitate and stearate was normalized to internal standard and cell number. Natural isotope abundance was corrected using IsoCor^58^.

### Cell survival assay

sh*ACSS1*/*2* or sh*scr* HepG2 stable cells were plated in 96-well plates at 1.5 × 10^4^ per well in 200 μl of medium treated with indicated conditions. The cell viability was measured after 48 h treatment by Cell Counting Kit-8 (Beyotime Biotechnology) according to the manufacturer's instructions. CCK-8 solution was incubated with cells for 1 h at 37 °C. Absorbances at 450 nm were collected by ELx800 Universal Microplate Reader (BioTek).

### Matrigel assay

Matrigel assay was performed as previously reported with minor modification^59^. Briefly, sh*scr* or sh*ACSS1/2* HepG2 cells were plated in 96-well plates (at 5,000 per well) precoated with 100% Matrigel bed and cultured in DMEM supplemented with 10% FBS, and 2% Matrigel for 12 days. The fresh culture media was exchanged every 2 days. The spheroids were monitored by microscopy. Spheroid size was determined at the times indicated by automated imaging on an inverted microscope (Olympus Fluorescence Microscope IX18).

### Hepatocarcinoma cancer samples

Hepatocarcinoma cancer samples were obtained from the 10th People's Hospital, Shanghai (affiliated to Tongji University), with consents from the patients. The procedure related to human subjects was approved by the Ethics Committee of the Institutes of Biomedical Sciences (IBS), Fudan University. Direct immunoblotting was performed as mentioned above.

### IHC staining

Human HCC sections were de-paraffinized in xylene and hydrated in graded ethanol, followed by deionized water. Endogenous peroxidase activity was inactivated with 3% hydrogen peroxidase in methanol for 30 min. Antigen retrieval was performed by boiling the sections in 0.01 M citrate buffer (pH 6.0) for 25 min in microwave oven. The sections were incubated with normal goat serum for 20 min to block non-specific staining and then incubated overnight at 4 °C with primary antibodies. After incubation for 45 min with a HRP-conjugated anti-rabbit secondary antibody, the sections were detected using diaminobenzidine (DAB) kit according to the manufacturer's instructions, followed by counterstaining with hematoxylin. The primary antibodies were used as follows: anti-H3K9 acetyl (ab32129, Abcam, 1:150 dilution), anti-H3K27 acetyl (ab45173, Abcam, 1:80 dilution), anti-H3K56 acetyl (ab76307, Abcam, 1:60 dilution), anti-ACSS2 (ab133664, Abcam, 1:50 dilution), anti-ACSS1 antibody (HPA041014, Sigma, 1:50 dilution), and anti-FASN (C-20140, Santa Cruz, 1:40 dilution). The negative controls were performed by omitting the primary antibodies.

### Statistical analysis

All data were statistically analysed with two-tailed unpaired Student's *t*-test. The correlation analysis between *FASN* and *ACSS1/2* mRNA levels in 190 human HCC samples from TCGA data base were carried out by using Pearson's coefficient test and the correlation analysis between histone acetylation and FASN expression in human HCC samples by Spearman's correlation test. The values of *P*<0.05 were considered as statistically significant.

### Data availability

The analysis result in Supplementary Fig. 6a is based upon data generated by the TCGA Research Network: http://cancergenome.nih.gov/ Data supporting the findings of this study are available within the article and its Supplementary Information files, and from the corresponding author upon request.

## Additional information

**How to cite this article:** Gao, X. *et al.* Acetate functions as an epigenetic metabolite to promote lipid synthesis under hypoxia. *Nat. Commun.* 7:11960 doi: 10.1038/ncomms11960 (2016).

## Supplementary Material

Supplementary Information Supplementary Figures 1-7 and Supplementary Table 1
