# Supplementary material for: Acetate functions as an epigenetic metabolite to promote lipid synthesis under hypoxia
Source: Nat Commun. 2016 Jun 30;7:11960. doi: 10.1038/ncomms11960 (PMC4931325; doi:10.1038/ncomms11960)
Supplement: Supplementary Information — Supplementary Figures 1-7 and Supplementary Table 1 [file ncomms11960-s1.pdf]

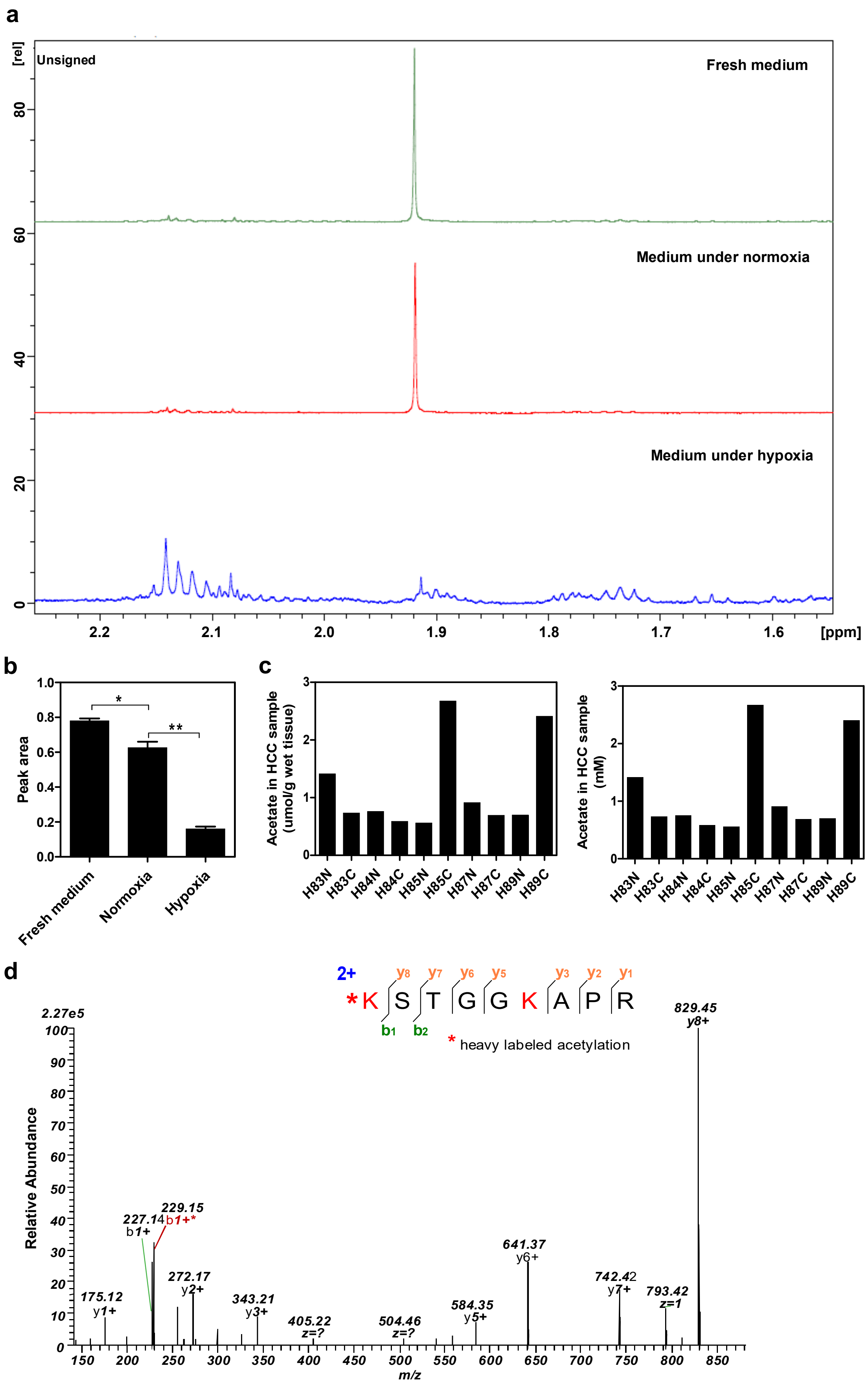
a
[rel]
Unsigned
Fresh medium
Medium under normoxia
Medium under hypoxia
80
60
40
20
0
2.2
2.1
2.0
1.9
1.8
1.7
1.6
[ppm]
b
Peak area
1.0
0.8
0.6
0.4
0.2
0.0
*
**
Fresh medium
Normoxia
Hypoxia
c
Acetate in HCC sample (umol/g wet tissue)
Acetate in HCC sample (mM)
H83N
H83C
H84N
H84C
H85N
H85C
H87N
H87C
H89N
H89C
d
2+
*K S T G G K A P R
* heavy labeled acetylation
Relative Abundance
m/z
2.27e5
829.45
y8+
227.14
b1+
229.15
b1+*
175.12
y1+
272.17
y2+
343.21
y3+
405.22
z=?
504.46
z=?
584.35
y5+
641.37
y6+
742.42
y7+
793.42
z=1

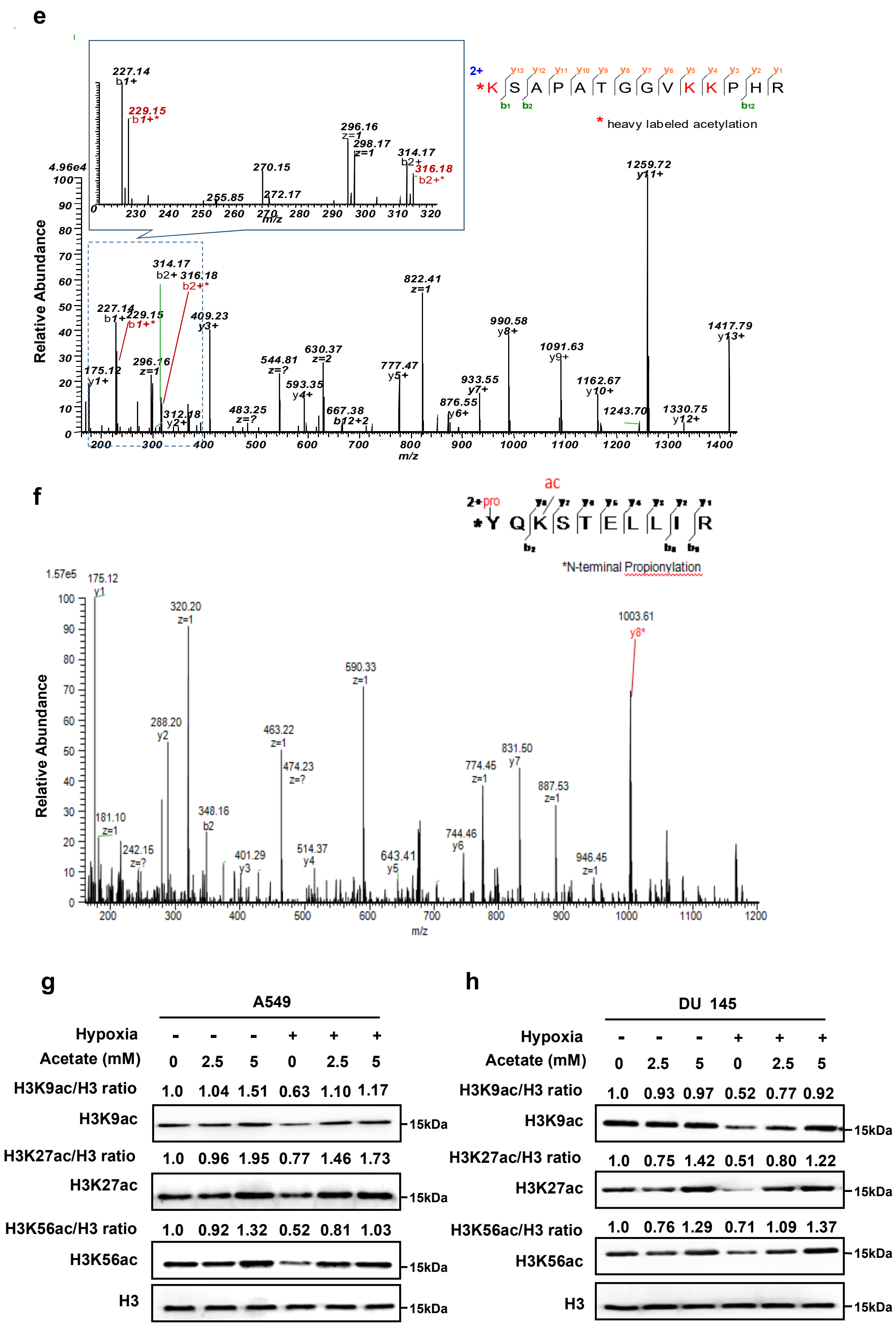
e
2+
*K S A P A T G G V K K P H R
* heavy labeled acetylation
Relative Abundance
m/z
1259.72
y11+
1417.79
y13+
822.41
z=1
990.58
y8+
1091.63
y9+
f
ac
2+pro
*Y Q K S T E L L I R
*N-terminal Propionylation
Relative Abundance
m/z
1003.61
y8*
590.33
z=1
320.20
z=1
175.12
y1
g
A549
Hypoxia - - - + + +
Acetate (mM) 0 2.5 5 0 2.5 5
H3K9ac/H3 ratio 1.0 1.04 1.51 0.63 1.10 1.17
H3K9ac
H3K27ac/H3 ratio 1.0 0.96 1.95 0.77 1.46 1.73
H3K27ac
H3K56ac/H3 ratio 1.0 0.92 1.32 0.52 0.81 1.03
H3K56ac
H3
15kDa
h
DU 145
Hypoxia - - - + + +
Acetate (mM) 0 2.5 5 0 2.5 5
H3K9ac/H3 ratio 1.0 0.93 0.97 0.52 0.77 0.92
H3K9ac
H3K27ac/H3 ratio 1.0 0.75 1.42 0.51 0.80 1.22
H3K27ac
H3K56ac/H3 ratio 1.0 0.76 1.29 0.71 1.09 1.37
H3K56ac
H3
15kDa

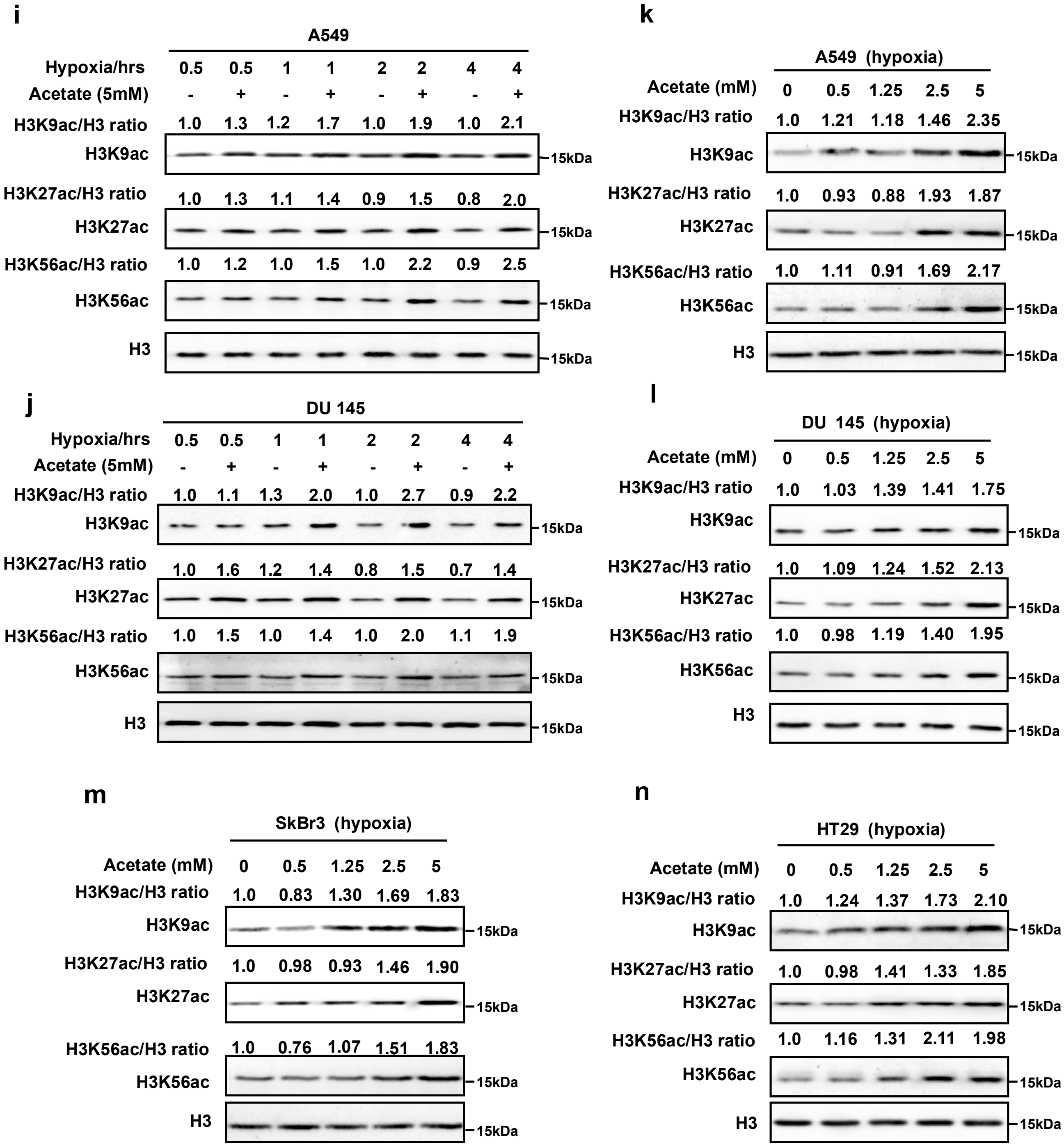

Supplementary Figure 1 Acetate rescues hypoxia-induced reduction of H3K9, H3K27 and H3K56 acetylation

(**a**) PLC-8024 cells were cultured in DMEM medium with 10% fetal bovine serum (FBS) for 24 hours. ^{1}H-NMR spectra of acetate (1.91 to 1.92 ppm at chemical shift) from fresh medium (green), medium of cancer cells cultured under normoxia (red) and medium of cancer cells cultured under hypoxia (blue). All of the peak areas were normalized with internal standard. (**b**) Peak areas of acetate ^{1}H-NMR spectra from fresh medium, medium of cancer cells cultured under normoxia and medium of

cancer cells cultured under hypoxia. The results were presented as mean ± s.d. of triplicate experiments (* $P<0.05$; ** $P<0.01$; by Student's *t*-test). (**c**) ^{1}H-NMR analysis of the acetate concentration in several human hepatocellular carcinoma (designated as C) and adjacent normal tissues (designated as N). Acetate (1.92 ppm in the spectrum) concentration was calculated by mole number of internal standard (TSP-d4, 0.00 ppm). (**d-f**) MS/MS spectra identified peptides with modification of $^{13}C_2$-labeled acetylation. The b and y ions for the identification of peptide were indicated in each spectrum, and product ion specific for the identification of $^{13}C_2$-acetylation were labeled in red. (d) MS/MS spectra of K(ac)STGGKAPR, (e) MS/MS spectra of K(ac)SAPASTGGVKKPHR, (f) MS/MS spectra of YQK(ac)STELLIR. Acetylation and propionylation sites were indicated in the peptide sequence. (**g-h**) A549 (g) and DU 145 (h) cells were treated with indicated concentrations of acetate under normoxia or hypoxia for 4 hours. Histone acetylation levels were determined by western blot. (**i-j**) Acetate increases H3K9, H3K27 and H3K56 acetylation levels in a time-dependent manner under hypoxia. A549 cells (i) and DU 145 cells (j) were treated with or without 5mM acetate for 0.5, 1, 2 and 4 hours under hypoxia, respectively. The global histone acetylation levels were determined by western blot. (**k-n**) Acetate increases H3K9, H3K27 and H3K56 acetylation levels in a dose-dependent manner under hypoxia. A549 (k), DU 145 (l), SkBr3 (m) and HT29 (n) cells were treated with indicated concentrations of acetate (0, 0.5, 1.25, 2.5, 5mM) for 4 hours under hypoxia. Histone acetylation levels were determined by western blot.

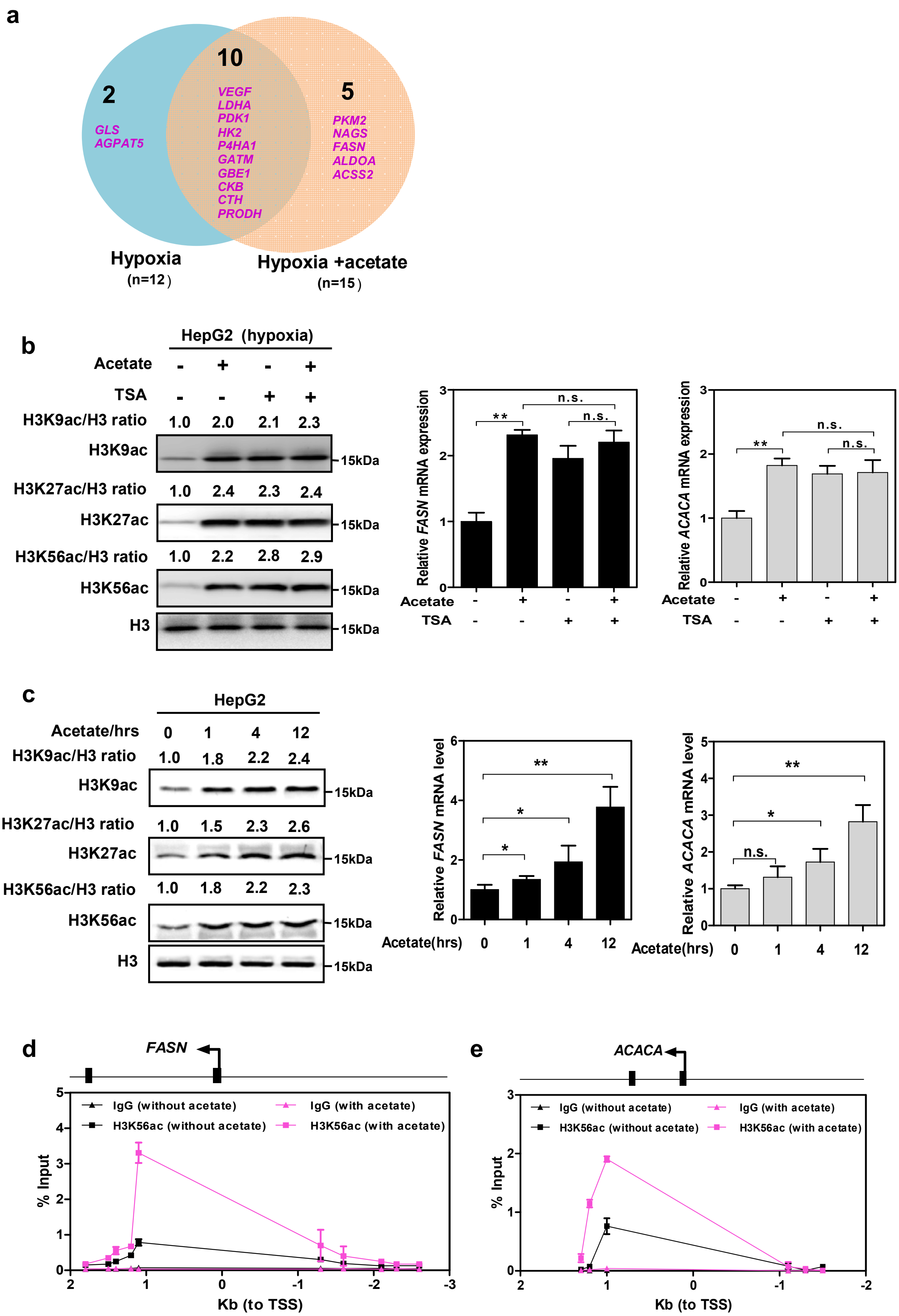

a
2
GLS
AGPAT5
10
VEGF
LDHA
PDK1
HK2
P4HA1
GATM
GBE1
CKB
CTH
PRODH
5
PKM2
NAGS
FASN
ALDOA
ACSS2
Hypoxia
(n=12)
Hypoxia +acetate
(n=15)
b
HepG2 (hypoxia)
Acetate - + - +
TSA - - + +
H3K9ac/H3 ratio 1.0 2.0 2.1 2.3
H3K9ac
15kDa
H3K27ac/H3 ratio 1.0 2.4 2.3 2.4
H3K27ac
H3K56ac/H3 ratio 1.0 2.2 2.8 2.9
H3K56ac
H3
Relative FASN mRNA expression
Relative ACACA mRNA expression
**
n.s.
c
HepG2
Acetate/hrs 0 1 4 12
H3K9ac/H3 ratio 1.0 1.8 2.2 2.4
H3K27ac/H3 ratio 1.0 1.5 2.3 2.6
H3K56ac/H3 ratio 1.0 1.8 2.2 2.3
Relative FASN mRNA level
Relative ACACA mRNA level
Acetate(hrs)
d
FASN
e
ACACA
IgG (without acetate)
IgG (with acetate)
H3K56ac (without acetate)
H3K56ac (with acetate)
% Input
Kb (to TSS)

f

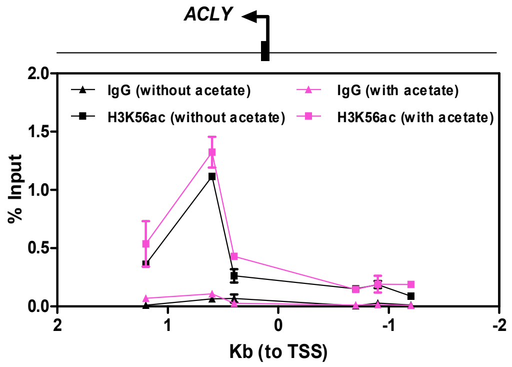
ACLY
IgG (without acetate)
H3K56ac (without acetate)
IgG (with acetate)
H3K56ac (with acetate)
% Input
Kb (to TSS)

g

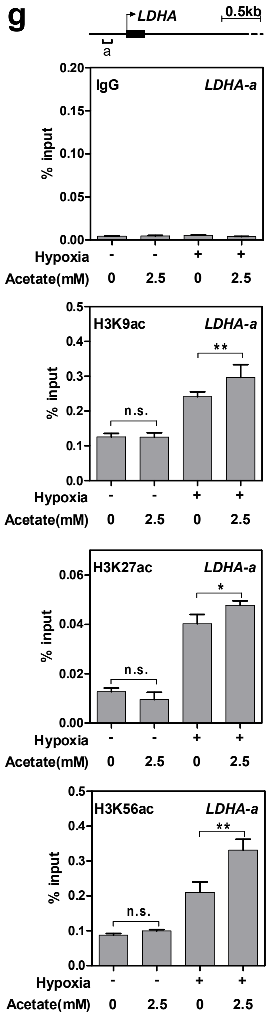
LDHA
0.5kb
a
IgG
LDHA-a
H3K9ac
H3K27ac
H3K56ac
% input
n.s.
**
*
Hypoxia
Acetate(mM)

h

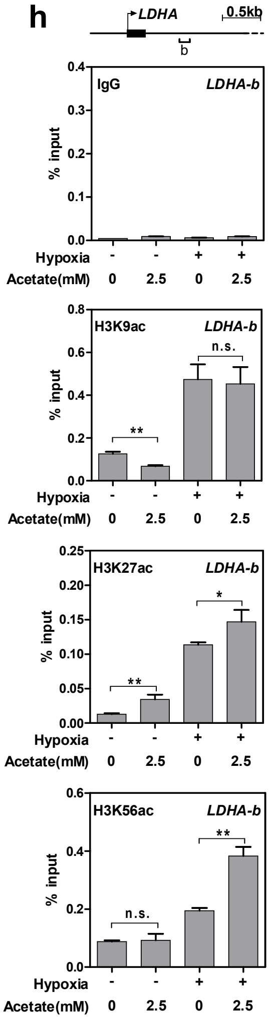
LDHA
0.5kb
b
IgG
LDHA-b
H3K9ac
H3K27ac
H3K56ac
% input
n.s.
**
*
Hypoxia
Acetate(mM)

i

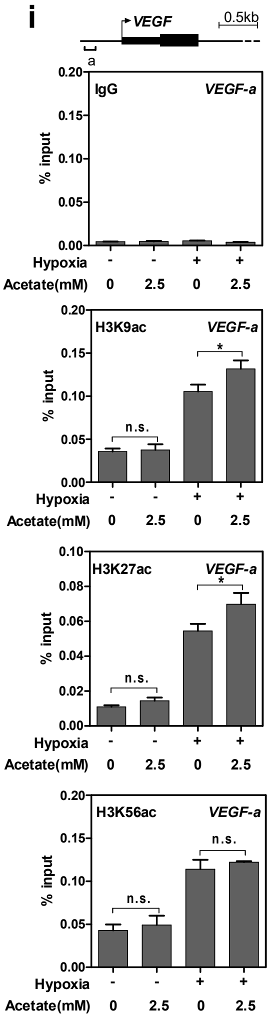
VEGF
0.5kb
a
IgG
VEGF-a
H3K9ac
H3K27ac
H3K56ac
% input
n.s.
*
Hypoxia
Acetate(mM)

j

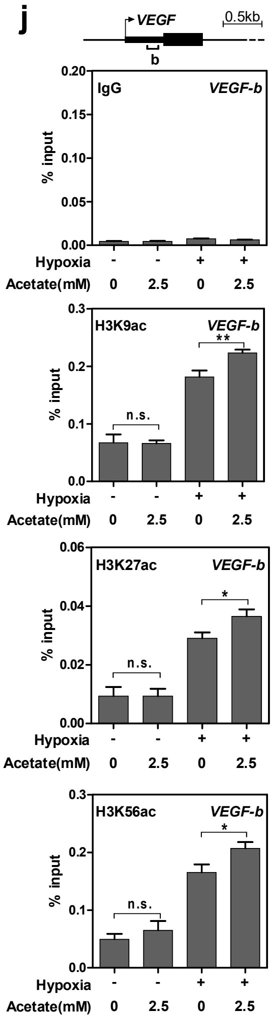
VEGF
0.5kb
b
IgG
VEGF-b
H3K9ac
H3K27ac
H3K56ac
% input
n.s.
**
*
Hypoxia
Acetate(mM)

k

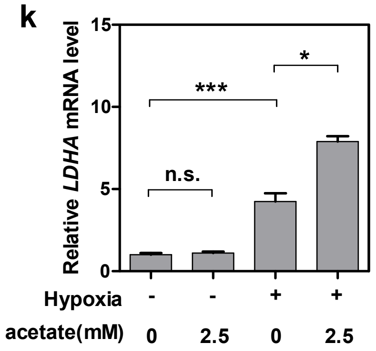
Relative LDHA mRNA level
***
*
n.s.
Hypoxia
acetate(mM)

l

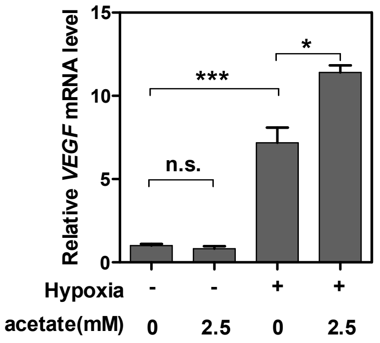
Relative VEGF mRNA level
***
*
n.s.
Hypoxia
acetate(mM)

Supplementary Figure 2 Acetate predominately activates lipid synthesis pathway through epigenetic regulation under hypoxia

(**a**) Venn diagram showed overlap of genes upregulated by 2-fold in hypoxia group and hypoxia with acetate group. The genes were highlighted in magenta. (**b**) HepG2 cells were treated with 2.5mM acetate and/or 0.5μM TSA under hypoxia for 12 hours. The histone acetylation levels were determined by western blot (left). *FASN* and *ACACA* mRNA levels were quantified by qPCR (right) and presented as mean ± s.d. of triplicate experiments (** P<0.01; n.s. means no significance; by Student's *t*-test). (**c**) Following exposure to hypoxia for 24 hours, HepG2 cells were treated with acetate for the indicated time under hypoxia. Histone acetylation levels were determined by western blot (left). *FASN* and *ACACA* mRNA levels were quantified by qPCR (right) and presented as mean ± s.d. of triplicate experiments (* P<0.05; ** P<0.01; n.s. means no significance; by Student's *t*-test). (**d-f**) HepG2 cells were treated with or without 5mM acetate under hypoxia for 4 hours. H3K56 acetylation level at the promoter region of *FASN* (d), *ACACA* (e), and *ACLY* (f) was determined by ChIP-qPCR. Rabbit IgG was included as a negative control. Arrow indicates the promoter orientation. The results were normalized to input and presented as mean ± s.d. of triplicate experiments. Primers were shown in Supplementary Table 1. (**g-j**) ChIP-qPCR assays showing H3K9, H3K27, and H3K56 acetylation levels at the promoter regions of *LDHA* (g-h) and *VEGF* (i-j) in HepG2 cells treated with or without 2.5mM acetate under normoxia or hypoxia for 4 hours. Two sets of primers for *LDHA* and *VEGF* (denoted by a and b) were used and shown in Supplementary Table 1. The results were presented as mean ± s.d. of triplicate experiments (*p<0.05; **p<0.01; n.s. means no significance; by Student's *t*-test). (**k-l**) *LDHA* and *VEGF* mRNA levels in HepG2 cells treated with or without 2.5mM acetate for 12 hours under normoxia or hypoxia were quantified by qPCR. The results were presented as mean ± s.d. of triplicate experiments (*p<0.05; ***p<0.01; n.s. means no significance; by Student's *t*-test).

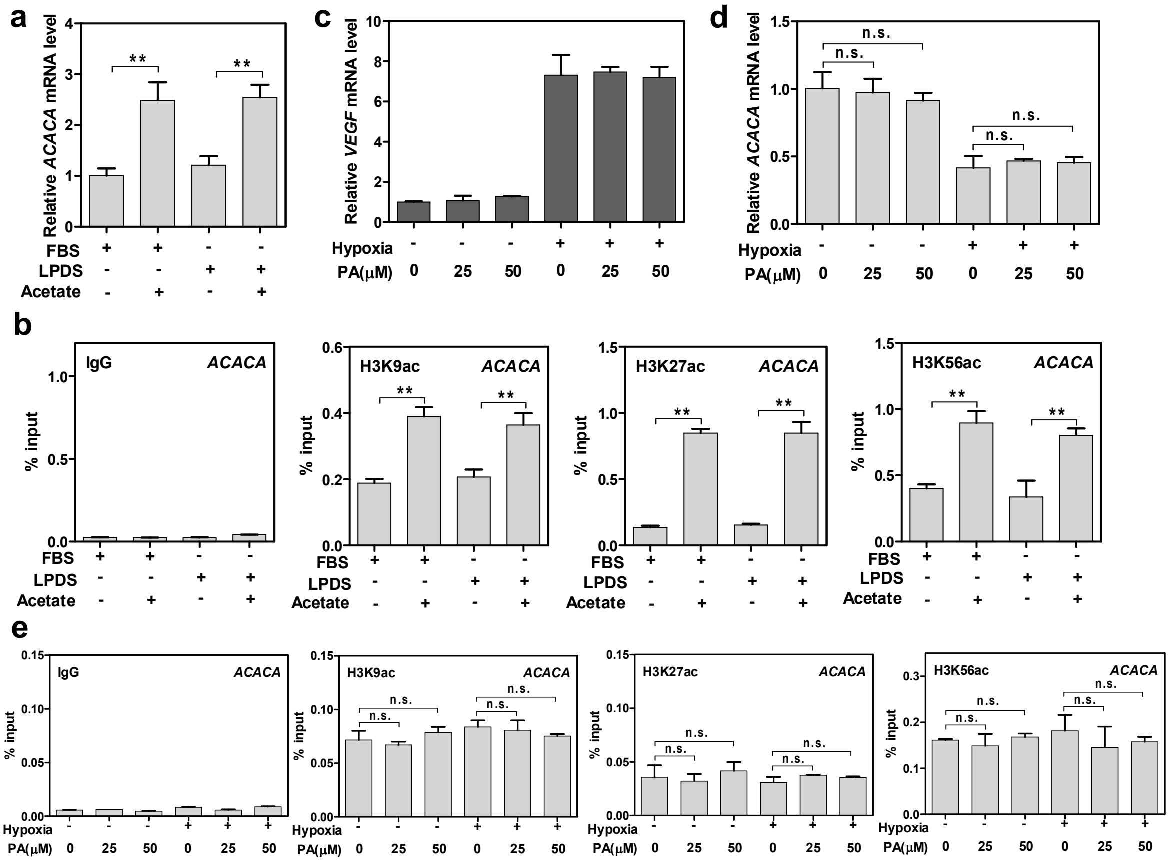

Supplementary Figure 3 Acetate epigenetically activates lipogenic genes without reflecting cellular lipid demands

(**a**) *ACACA* mRNA expression in HepG2 cells treated with or without 2.5 mM acetate in media supplemented with 10%FBS or 10% lipid-depleted serum (LPDS) for 12 hours under hypoxia (1% O_2) by qPCR. The results were presented as mean ± s.d. of triplicate experiments (**p<0.01; by Student's *t*-test). (**b**) ChIP-qPCR assays showing H3K9, H3K27, and H3K56 acetylation levels at *ACACA* promoter region in HepG2 cells treated with or without 2.5 mM acetate in media supplemented with 10% FBS or 10% LPDS for 4 hours under hypoxia. The results were normalized to input and presented as mean ± s.d. of triplicate experiments (**p<0.01; by Student's *t*-test). (**c-d**) *VEGF* (c) and *ACACA* (d) mRNA expressions in HepG2 cells treated with indicated concentrations of palmitate (PA) in media supplemented with 10% LPDS for 12 hours under normoxia or hypoxia (1% O_2). The results were detected by qPCR and presented as mean ± s.d. of triplicate experiments (n.s. means no significance; by Student's *t*-test). (**e**) ChIP-qPCR assays showing H3K9, H3K27, and H3K56 acetylation levels at *ACACA* promoter region in HepG2 cells treated with indicated concentrations of PA in media supplemented with 10% LPDS for 4 hours under normoxia or hypoxia. The results were normalized to input and presented as mean ± s.d. of triplicate experiments (n.s. means no significance; by Student's *t*-test).

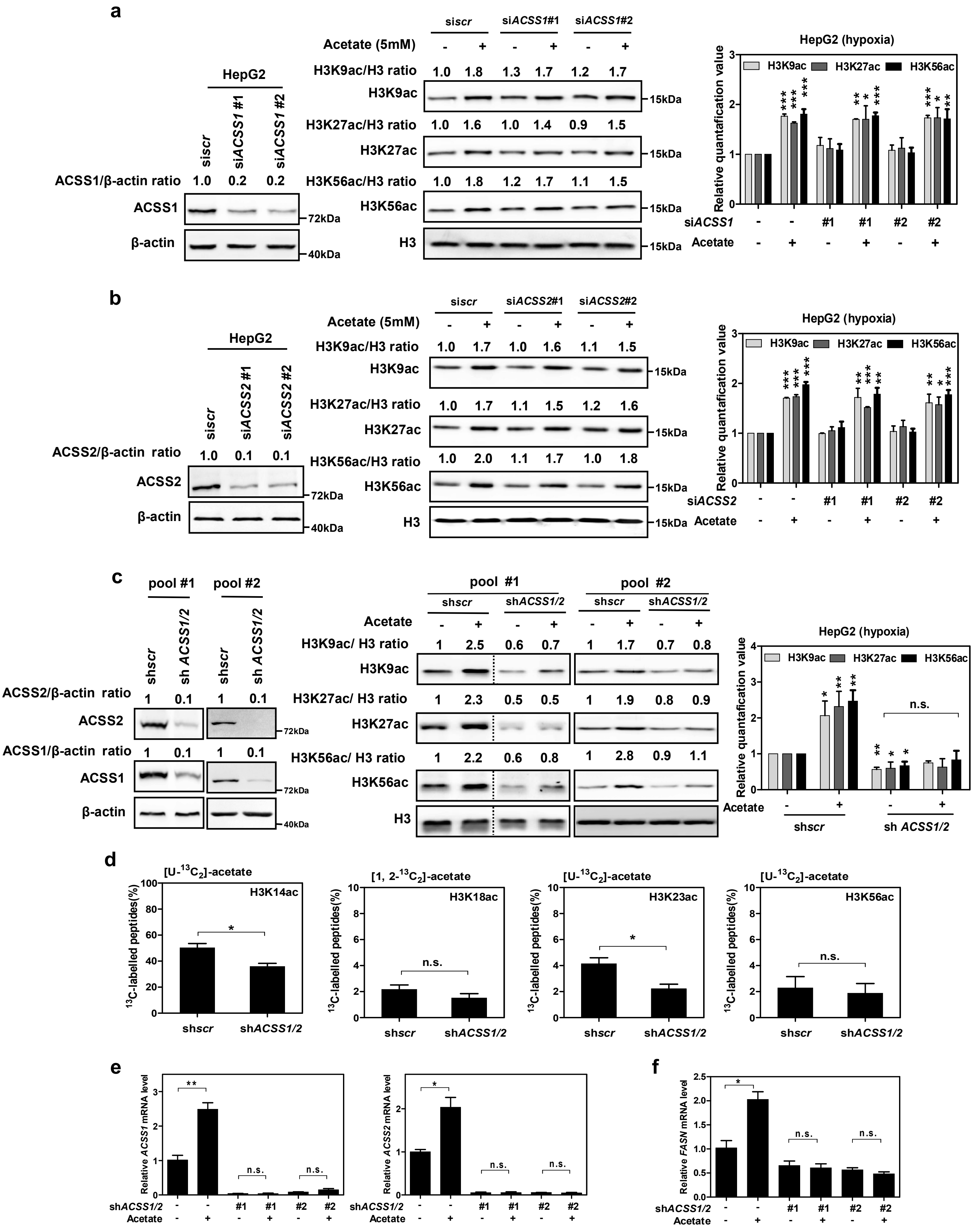

a
HepG2
siscr
siACSS1 #1
siACSS1 #2
ACSS1/β-actin ratio 1.0 0.2 0.2
ACSS1
β-actin
72kDa
40kDa
siscr
siACSS1#1
siACSS1#2
Acetate (5mM) - + - + - +
H3K9ac/H3 ratio 1.0 1.8 1.3 1.7 1.2 1.7
H3K9ac
H3K27ac/H3 ratio 1.0 1.6 1.0 1.4 0.9 1.5
H3K27ac
H3K56ac/H3 ratio 1.0 1.8 1.2 1.7 1.1 1.5
H3K56ac
H3
15kDa
HepG2 (hypoxia)
H3K9ac
H3K27ac
H3K56ac
Relative quantafication value
siACSS1 - - #1 #1 #2 #2
Acetate - + - + - +
b
HepG2
siscr
siACSS2 #1
siACSS2 #2
ACSS2/β-actin ratio 1.0 0.1 0.1
ACSS2
β-actin
72kDa
40kDa
siscr
siACSS2#1
siACSS2#2
Acetate (5mM) - + - + - +
H3K9ac/H3 ratio 1.0 1.7 1.0 1.6 1.1 1.5
H3K9ac
H3K27ac/H3 ratio 1.0 1.7 1.1 1.5 1.2 1.6
H3K27ac
H3K56ac/H3 ratio 1.0 2.0 1.1 1.7 1.0 1.8
H3K56ac
H3
15kDa
HepG2 (hypoxia)
H3K9ac
H3K27ac
H3K56ac
Relative quantafication value
siACSS2 - - #1 #1 #2 #2
Acetate - + - + - +
c
pool #1
pool #2
shscr
sh ACSS1/2
ACSS2/β-actin ratio 1 0.1 1 0.1
ACSS2
ACSS1/β-actin ratio 1 0.1 1 0.1
ACSS1
β-actin
72kDa
40kDa
pool #1
pool #2
shscr
shACSS1/2
Acetate - + - + - + - +
H3K9ac/ H3 ratio 1 2.5 0.6 0.7 1 1.7 0.7 0.8
H3K9ac
H3K27ac/ H3 ratio 1 2.3 0.5 0.5 1 1.9 0.8 0.9
H3K27ac
H3K56ac/ H3 ratio 1 2.2 0.6 0.8 1 2.8 0.9 1.1
H3K56ac
H3
HepG2 (hypoxia)
H3K9ac
H3K27ac
H3K56ac
Relative quantafication value
n.s.
Acetate - + - +
shscr
sh ACSS1/2
d
[U-13C2]-acetate
H3K14ac
[1, 2-13C2]-acetate
H3K18ac
[U-13C2]-acetate
H3K23ac
[U-13C2]-acetate
H3K56ac
13C-labelled peptides(%)
n.s.
shscr
shACSS1/2
e
Relative ACSS1 mRNA level
Relative ACSS2 mRNA level
n.s.
shACSS1/2 - - #1 #1 #2 #2
Acetate - + - + - +
f
Relative FASN mRNA level
n.s.
shACSS1/2 - - #1 #1 #2 #2
Acetate - + - + - +

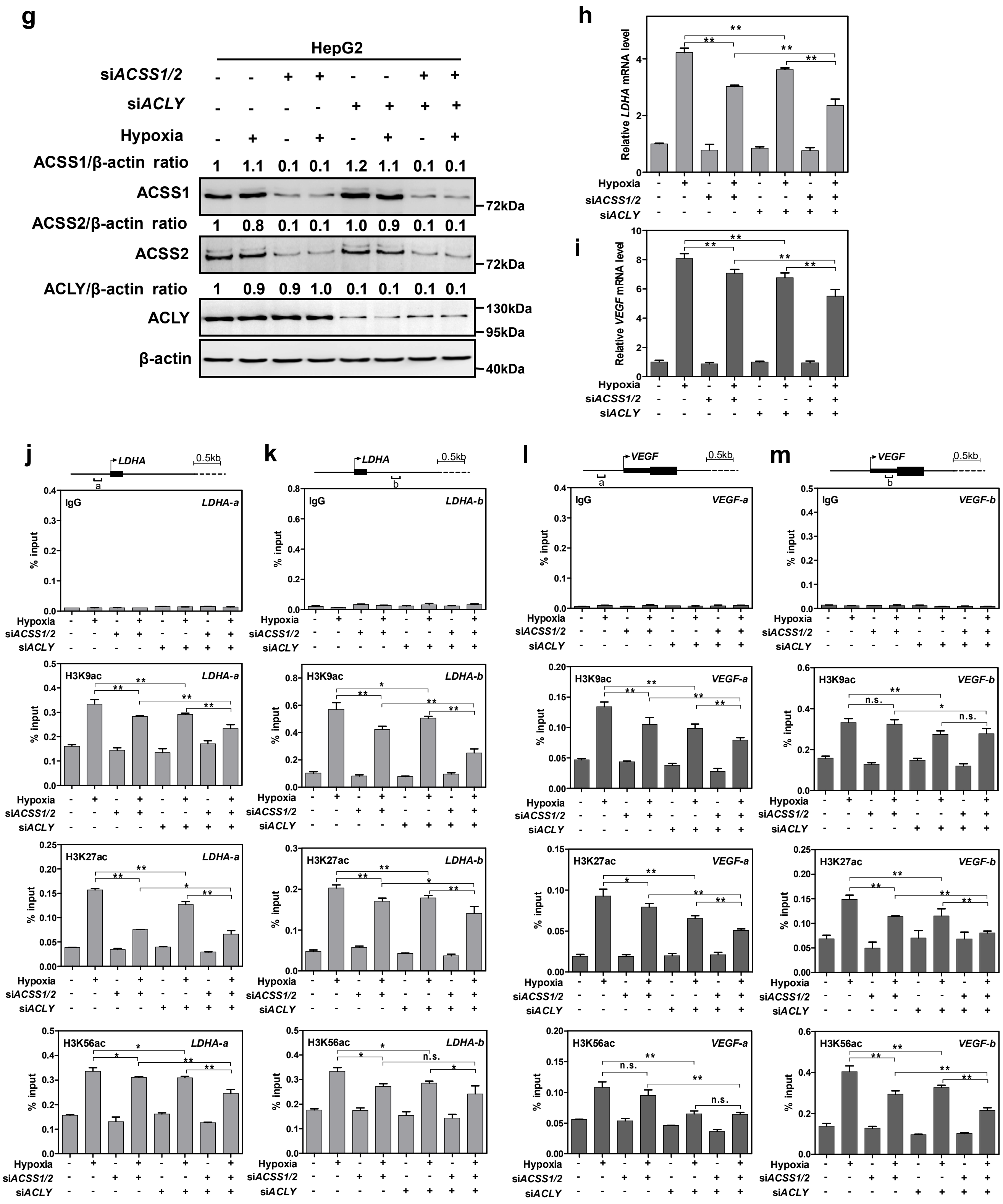

Supplementary Figure 4 Both ACSS1 and ACSS2 are involved in acetate-induced epigenetic regulation of *de novo* lipogenesis

(**a-b**) Histone acetylation in transient-knockdown either *ACSS1* (si*ACSS1*) (a) or *ACSS2* (si*ACSS2*) (b) HepG2 cells treated with or without acetate under hypoxia for 4 hours were analyzed by western blot compared to scramble (si*scr*). Two different

siRNAs targeting *ACSS1* or *ACSS2* were applied. The quantification analysis (right) was presented as mean ± s.d. of triplicate experiments (*p<0.05; **p<0.01; ***p<0.001; by Student's *t*-test). (**c**) Histone acetylation levels in scramble (sh*scr*) or stably double-knockdown *ACSS1* and *ACSS2* (sh*ACSS1/2*) HepG2 cells treated as in panel (a) were analyzed with western blot (middle). Two different stable cell pools generated from two different sets of shRNAs targeting *ACSS1/2* were used. The quantification analysis (right) was presented as mean ± s.d. of triplicate experiments (*p<0.05; **p<0.01; n.s. means no significance; by Student's *t*-test). (**d**) Quantification of $^{13}C_2$-labeled acetylated histone H3 peptides in sh*scr* or sh*ACSS1/2* HepG2 cells treated with [U-$^{13}C_2$]-acetate for 4 hours under hypoxia was analyzed by LC-MRM-MS. The percentage indicates the ratio of H3K[$^{13}C_2$-ac]/total H3Kac in each site. The quantification analysis of histone acetylation levels were presented as mean ± s.d. of triplicate experiments (*p<0.05; n.s. means no significance; by Student's *t*-test). (**e-f**) *ACSS1*, *ACSS2*, and *FASN* mRNA levels in sh*scr* or sh*ACSS1/2* HepG2 cells treated with or without 2.5mM acetate for 4 hours under hypoxia following exposure to hypoxia for 24 hours were quantified by qPCR. The results were presented as mean ± s.d. of triplicate experiments (* P<0.05; ** P<0.01; n.s. means no significance; by Student's *t*-test). (**g**) The *ACSS1/2* and *ACLY* transient-knockdown efficiency was verified by western blot. (**h-i**) qPCR quantification of *LDHA* (h) and *VEGF* (i) mRNA levels in si*ACSS1/2*, si*ACLY*, and si*ACSS1/2+ACLY* HepG2 cells treated under normoxia or hypoxia for 12 hours. The results were presented as mean ± s.d. of triplicate experiments (**p<0.01; by Student's *t*-test). (**j-m**) Histone acetylation levels at *LDHA* (j-k) and *VEGF* (l-m) promoter regions in si*ACSS1/2*, si*ACLY*, and si*ACSS1/2+ACLY* HepG2 cells treated under normoxia or hypoxia for 4 hours were analyzed by ChIP-qPCR and presented as mean ± s.d. of triplicate experiments (*p<0.05; **p<0.01; n.s. means no significance; by Student's *t*-test).

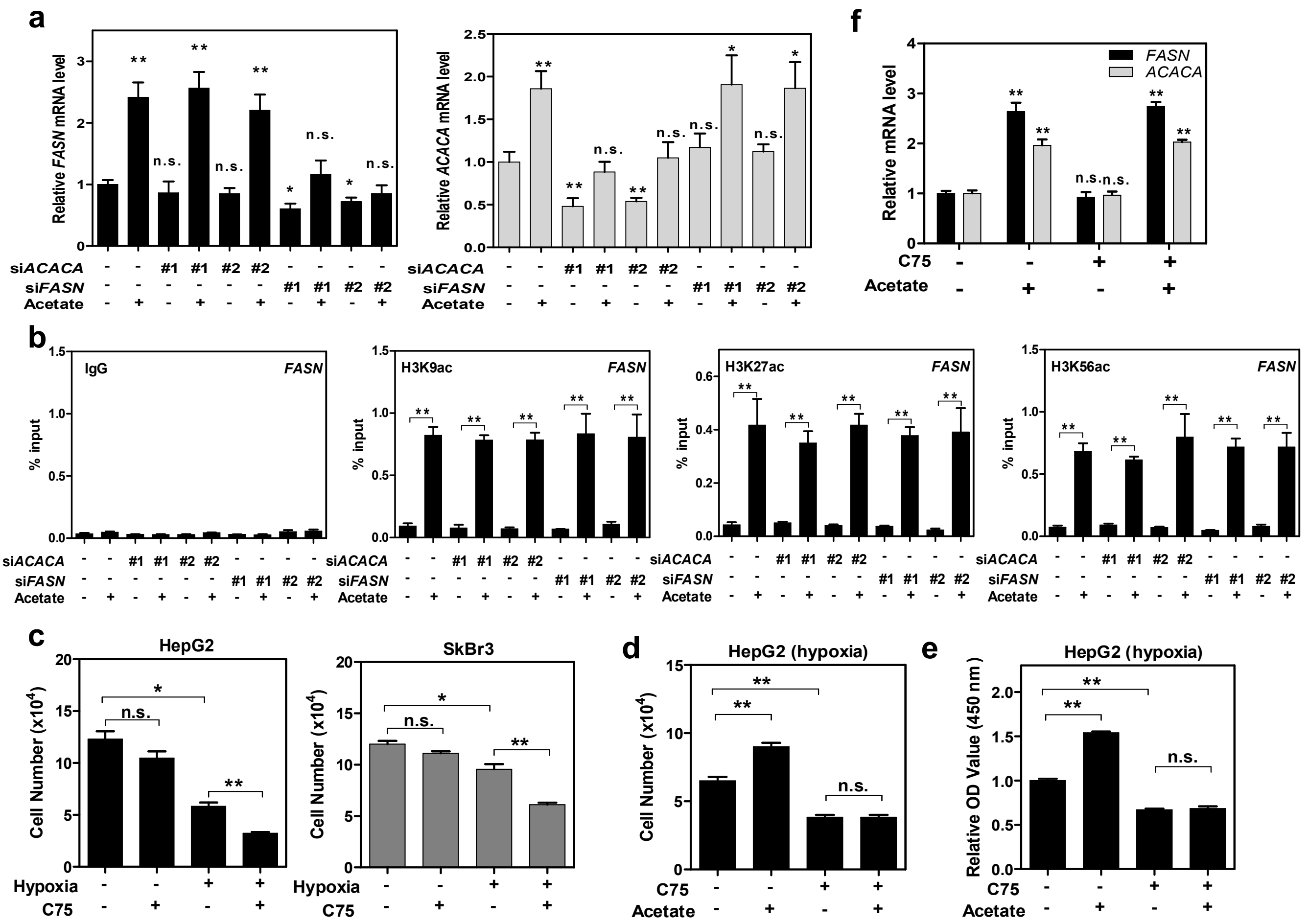

Supplementary Figure 5 Epigenetic regulation of de novo lipogenesis by acetate is vital for cell survival under hypoxic stress

(**a**) *FASN* (left) and *ACACA* (right) mRNA levels in scramble or transient-knockdown *FASN* or *ACACA* HepG2 cells treated with or without 2.5mM acetate for 12 hours under hypoxia after 60 hours siRNA transfection were analyzed by qPCR and presented as mean ± s.d. of triplicate experiments (*p<0.05; **p<0.01; n.s. means no significance; by Student's *t*-test). (**b**) ChIP-qPCR assays showing histone acetylation levels at *FASN* promoter region in scramble or si*FASN* or si*ACACA* HepG2 cells treated with or without 2.5mM acetate for 4 hours under hypoxia. Acetate treatment was carried out after 68 hours siRNA transfection. The results were normalized to input and presented as mean ± s.d. of triplicate experiments (**p<0.01; by Student's *t*-test). (**c**) Sensitivity of HepG2 (left) and SkBr3 (right) cells to C75 (2.5 mM) treatment under normoxia or hypoxia for 48 hours. The results were presented as mean ± s.d. of triplicate experiments (*p<0.05; **p<0.01; n.s. means no significance; by Student's *t*-test). (**d-e**) Viability of HepG2 cells treated with 2.5 mM acetate and/or C75 (2.5 mM) for 48 hours under hypoxia was determined via cell number counting (d) or CCK8 assay (e). The results were presented as mean ± s.d. of triplicate experiments (**p<0.01; n.s. means no significance; by Student's *t*-test). (**f**) *FASN* and *ACACA* mRNA levels in HepG2 cells treated as in panel (d) were analyzed by qPCR and presented as mean ± s.d. of triplicate experiments (**p<0.01; n.s. means no significance; by Student's *t*-test).

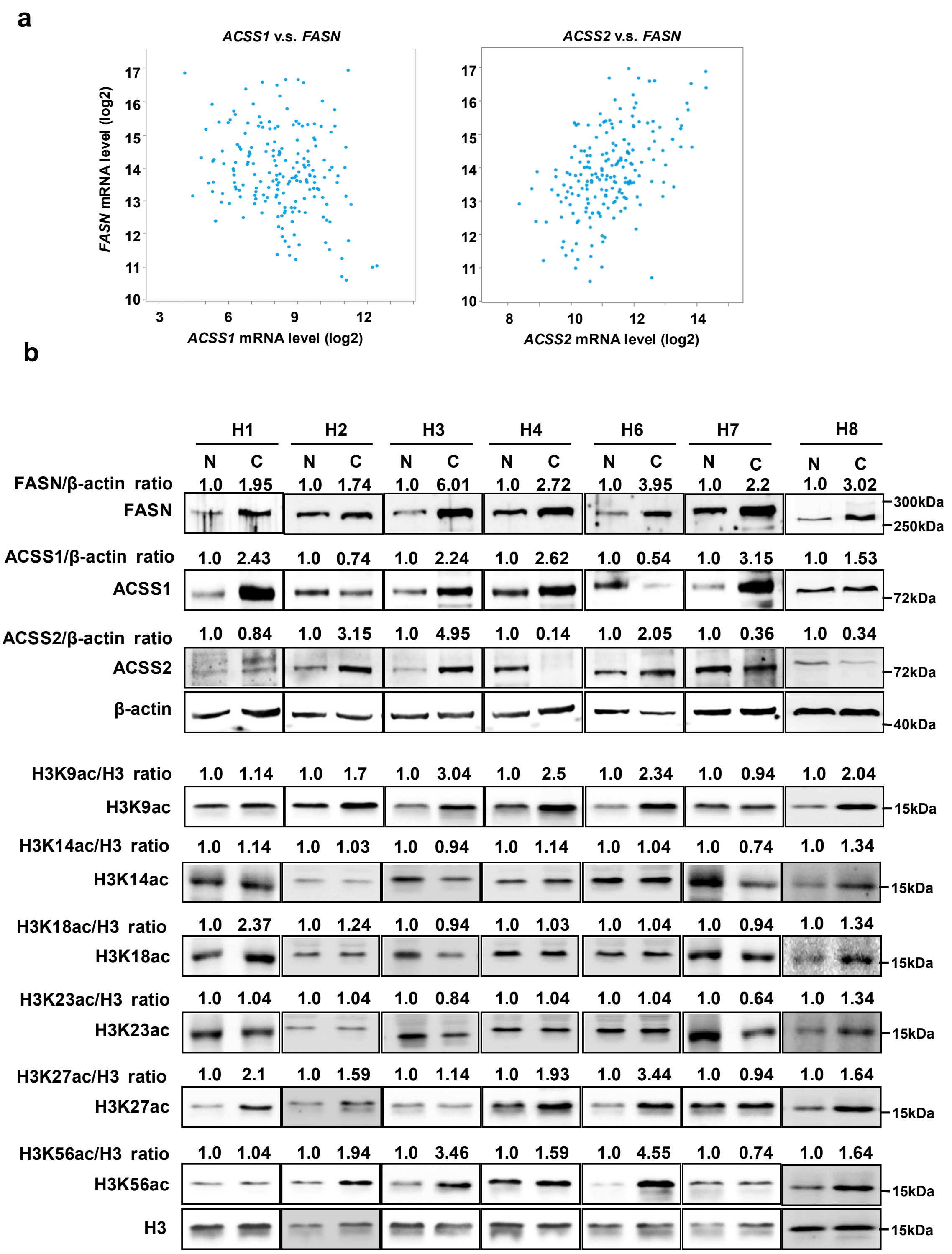
a
ACSS1 v.s. FASN
ACSS2 v.s. FASN
FASN mRNA level (log2)
17
16
15
14
13
12
11
10
3
6
9
12
ACSS1 mRNA level (log2)
8
10
12
14
ACSS2 mRNA level (log2)
b
H1 H2 H3 H4 H6 H7 H8
N C N C N C N C N C N C N C
FASN/β-actin ratio 1.0 1.95 1.0 1.74 1.0 6.01 1.0 2.72 1.0 3.95 1.0 2.2 1.0 3.02
FASN
300kDa
250kDa
ACSS1/β-actin ratio 1.0 2.43 1.0 0.74 1.0 2.24 1.0 2.62 1.0 0.54 1.0 3.15 1.0 1.53
ACSS1
72kDa
ACSS2/β-actin ratio 1.0 0.84 1.0 3.15 1.0 4.95 1.0 0.14 1.0 2.05 1.0 0.36 1.0 0.34
ACSS2
72kDa
β-actin
40kDa
H3K9ac/H3 ratio 1.0 1.14 1.0 1.7 1.0 3.04 1.0 2.5 1.0 2.34 1.0 0.94 1.0 2.04
H3K9ac
15kDa
H3K14ac/H3 ratio 1.0 1.14 1.0 1.03 1.0 0.94 1.0 1.14 1.0 1.04 1.0 0.74 1.0 1.34
H3K14ac
15kDa
H3K18ac/H3 ratio 1.0 2.37 1.0 1.24 1.0 0.94 1.0 1.03 1.0 1.04 1.0 0.94 1.0 1.34
H3K18ac
15kDa
H3K23ac/H3 ratio 1.0 1.04 1.0 1.04 1.0 0.84 1.0 1.04 1.0 1.04 1.0 0.64 1.0 1.34
H3K23ac
15kDa
H3K27ac/H3 ratio 1.0 2.1 1.0 1.59 1.0 1.14 1.0 1.93 1.0 3.44 1.0 0.94 1.0 1.64
H3K27ac
15kDa
H3K56ac/H3 ratio 1.0 1.04 1.0 1.94 1.0 3.46 1.0 1.59 1.0 4.55 1.0 0.74 1.0 1.64
H3K56ac
15kDa
H3
15kDa

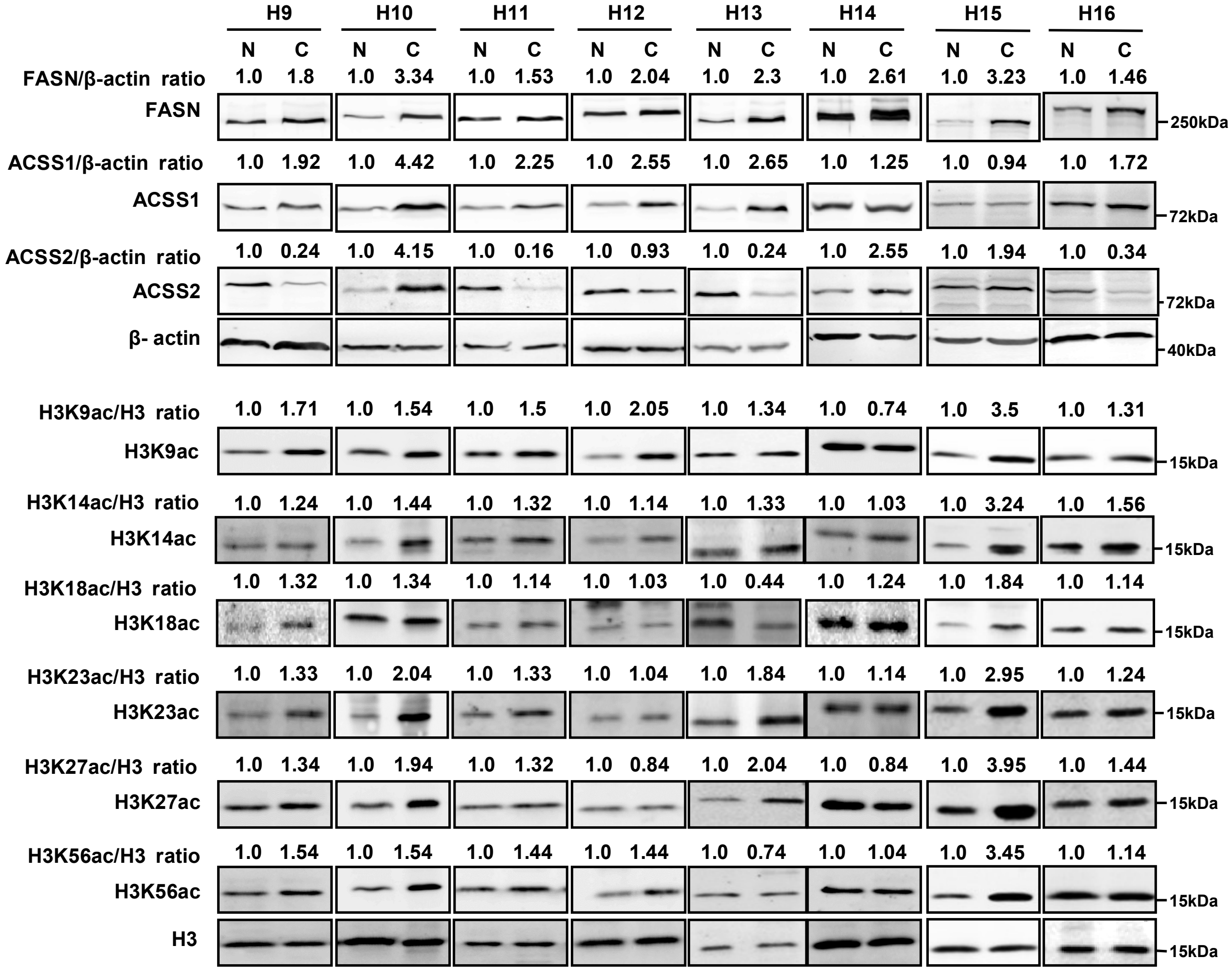
H9
H10
H11
H12
H13
H14
H15
H16
N C N C N C N C N C N C N C N C
FASN/β-actin ratio 1.0 1.8 1.0 3.34 1.0 1.53 1.0 2.04 1.0 2.3 1.0 2.61 1.0 3.23 1.0 1.46
FASN
250kDa
ACSS1/β-actin ratio 1.0 1.92 1.0 4.42 1.0 2.25 1.0 2.55 1.0 2.65 1.0 1.25 1.0 0.94 1.0 1.72
ACSS1
72kDa
ACSS2/β-actin ratio 1.0 0.24 1.0 4.15 1.0 0.16 1.0 0.93 1.0 0.24 1.0 2.55 1.0 1.94 1.0 0.34
ACSS2
72kDa
β- actin
40kDa
H3K9ac/H3 ratio 1.0 1.71 1.0 1.54 1.0 1.5 1.0 2.05 1.0 1.34 1.0 0.74 1.0 3.5 1.0 1.31
H3K9ac
15kDa
H3K14ac/H3 ratio 1.0 1.24 1.0 1.44 1.0 1.32 1.0 1.14 1.0 1.33 1.0 1.03 1.0 3.24 1.0 1.56
H3K14ac
15kDa
H3K18ac/H3 ratio 1.0 1.32 1.0 1.34 1.0 1.14 1.0 1.03 1.0 0.44 1.0 1.24 1.0 1.84 1.0 1.14
H3K18ac
15kDa
H3K23ac/H3 ratio 1.0 1.33 1.0 2.04 1.0 1.33 1.0 1.04 1.0 1.84 1.0 1.14 1.0 2.95 1.0 1.24
H3K23ac
15kDa
H3K27ac/H3 ratio 1.0 1.34 1.0 1.94 1.0 1.32 1.0 0.84 1.0 2.04 1.0 0.84 1.0 3.95 1.0 1.44
H3K27ac
15kDa
H3K56ac/H3 ratio 1.0 1.54 1.0 1.54 1.0 1.44 1.0 1.44 1.0 0.74 1.0 1.04 1.0 3.45 1.0 1.14
H3K56ac
15kDa
H3
15kDa

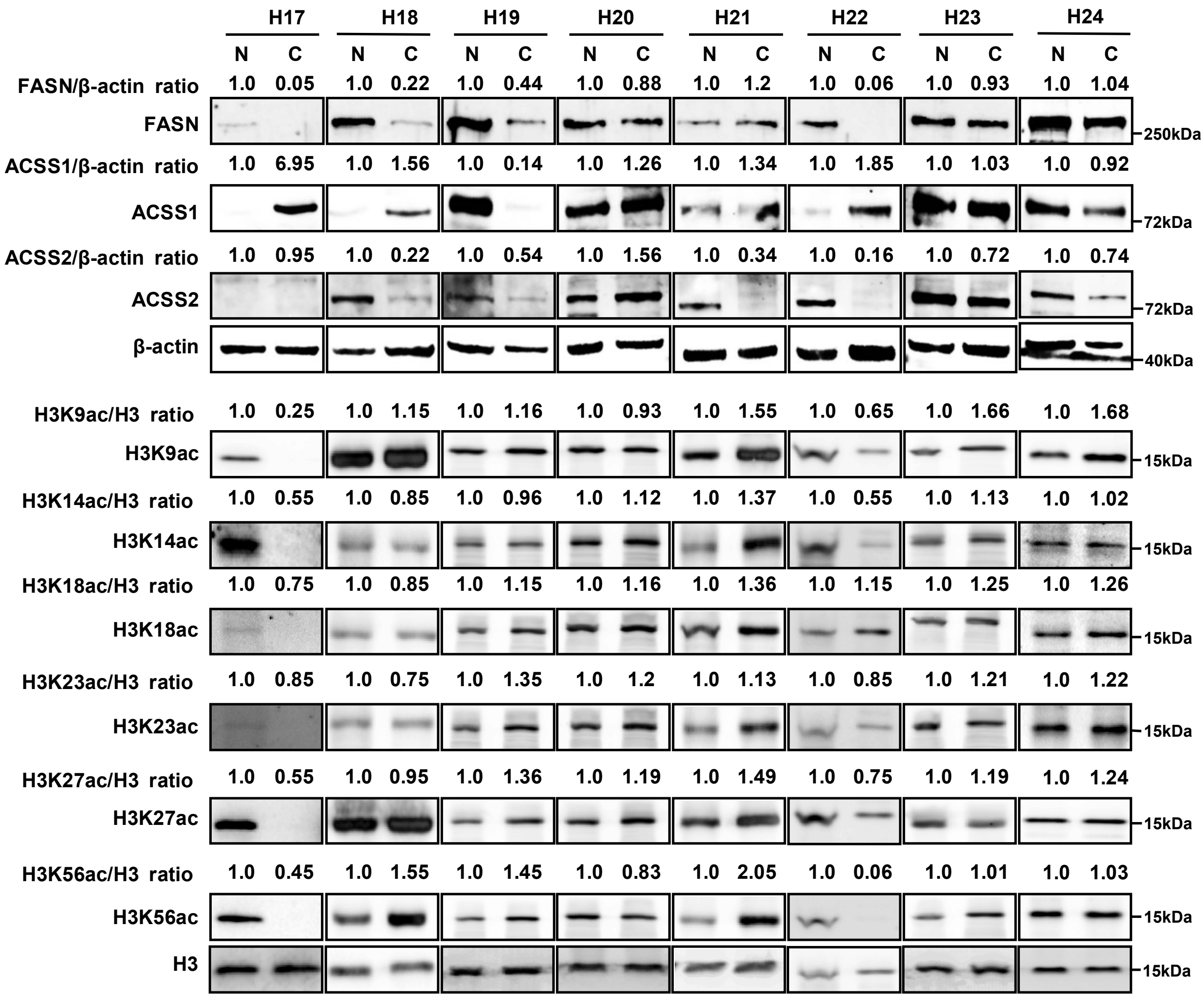
H17
H18
H19
H20
H21
H22
H23
H24
N C
FASN/β-actin ratio 1.0 0.05 1.0 0.22 1.0 0.44 1.0 0.88 1.0 1.2 1.0 0.06 1.0 0.93 1.0 1.04
FASN
250kDa
ACSS1/β-actin ratio 1.0 6.95 1.0 1.56 1.0 0.14 1.0 1.26 1.0 1.34 1.0 1.85 1.0 1.03 1.0 0.92
ACSS1
72kDa
ACSS2/β-actin ratio 1.0 0.95 1.0 0.22 1.0 0.54 1.0 1.56 1.0 0.34 1.0 0.16 1.0 0.72 1.0 0.74
ACSS2
72kDa
β-actin
40kDa
H3K9ac/H3 ratio 1.0 0.25 1.0 1.15 1.0 1.16 1.0 0.93 1.0 1.55 1.0 0.65 1.0 1.66 1.0 1.68
H3K9ac
15kDa
H3K14ac/H3 ratio 1.0 0.55 1.0 0.85 1.0 0.96 1.0 1.12 1.0 1.37 1.0 0.55 1.0 1.13 1.0 1.02
H3K14ac
15kDa
H3K18ac/H3 ratio 1.0 0.75 1.0 0.85 1.0 1.15 1.0 1.16 1.0 1.36 1.0 1.15 1.0 1.25 1.0 1.26
H3K18ac
15kDa
H3K23ac/H3 ratio 1.0 0.85 1.0 0.75 1.0 1.35 1.0 1.2 1.0 1.13 1.0 0.85 1.0 1.21 1.0 1.22
H3K23ac
15kDa
H3K27ac/H3 ratio 1.0 0.55 1.0 0.95 1.0 1.36 1.0 1.19 1.0 1.49 1.0 0.75 1.0 1.19 1.0 1.24
H3K27ac
15kDa
H3K56ac/H3 ratio 1.0 0.45 1.0 1.55 1.0 1.45 1.0 0.83 1.0 2.05 1.0 0.06 1.0 1.01 1.0 1.03
H3K56ac
15kDa
H3
15kDa

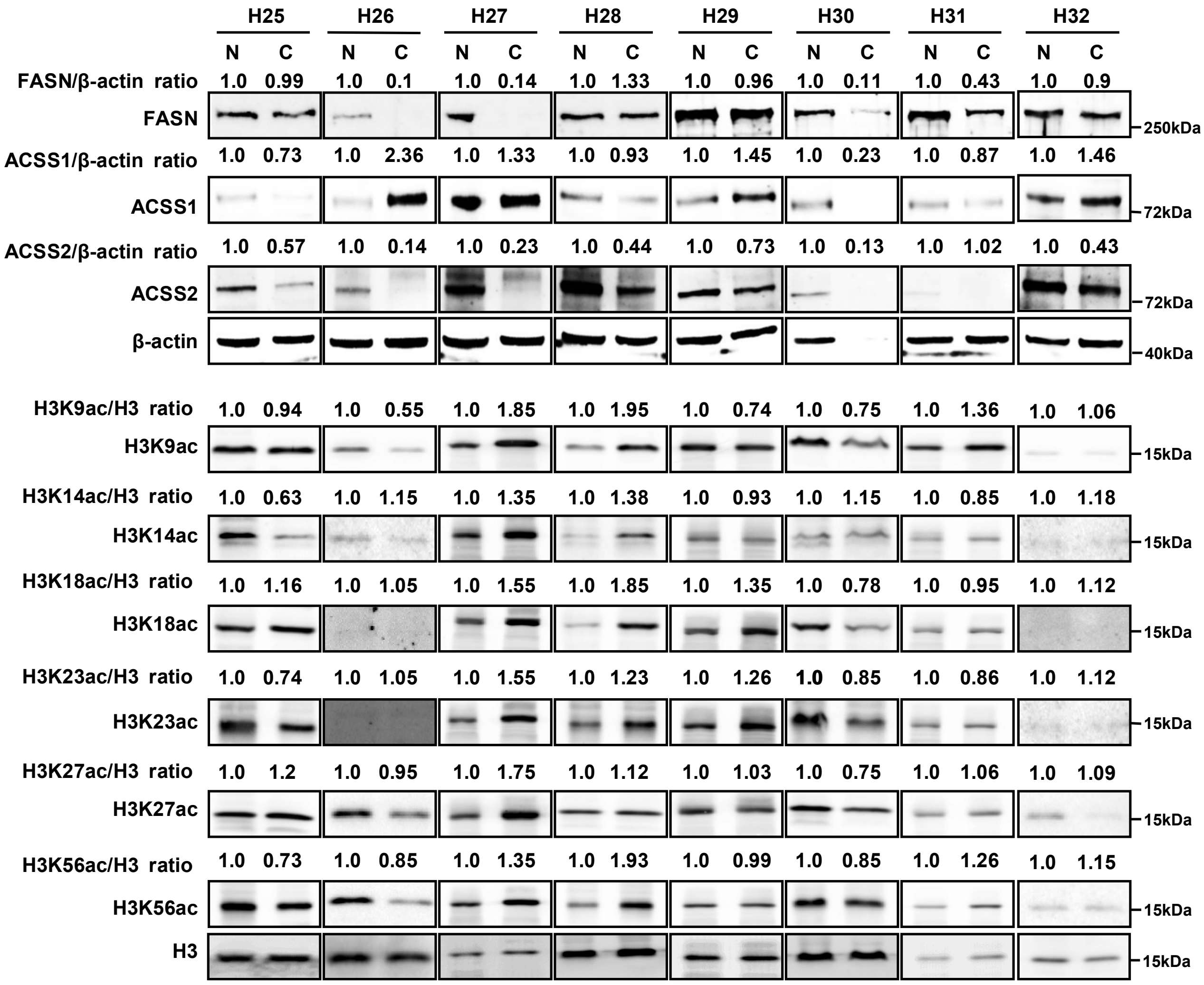
H25 H26 H27 H28 H29 H30 H31 H32
N C N C N C N C N C N C N C N C
FASN/β-actin ratio 1.0 0.99 1.0 0.1 1.0 0.14 1.0 1.33 1.0 0.96 1.0 0.11 1.0 0.43 1.0 0.9
FASN
250kDa
ACSS1/β-actin ratio 1.0 0.73 1.0 2.36 1.0 1.33 1.0 0.93 1.0 1.45 1.0 0.23 1.0 0.87 1.0 1.46
ACSS1
72kDa
ACSS2/β-actin ratio 1.0 0.57 1.0 0.14 1.0 0.23 1.0 0.44 1.0 0.73 1.0 0.13 1.0 1.02 1.0 0.43
ACSS2
72kDa
β-actin
40kDa
H3K9ac/H3 ratio 1.0 0.94 1.0 0.55 1.0 1.85 1.0 1.95 1.0 0.74 1.0 0.75 1.0 1.36 1.0 1.06
H3K9ac
15kDa
H3K14ac/H3 ratio 1.0 0.63 1.0 1.15 1.0 1.35 1.0 1.38 1.0 0.93 1.0 1.15 1.0 0.85 1.0 1.18
H3K14ac
15kDa
H3K18ac/H3 ratio 1.0 1.16 1.0 1.05 1.0 1.55 1.0 1.85 1.0 1.35 1.0 0.78 1.0 0.95 1.0 1.12
H3K18ac
15kDa
H3K23ac/H3 ratio 1.0 0.74 1.0 1.05 1.0 1.55 1.0 1.23 1.0 1.26 1.0 0.85 1.0 0.86 1.0 1.12
H3K23ac
15kDa
H3K27ac/H3 ratio 1.0 1.2 1.0 0.95 1.0 1.75 1.0 1.12 1.0 1.03 1.0 0.75 1.0 1.06 1.0 1.09
H3K27ac
15kDa
H3K56ac/H3 ratio 1.0 0.73 1.0 0.85 1.0 1.35 1.0 1.93 1.0 0.99 1.0 0.85 1.0 1.26 1.0 1.15
H3K56ac
15kDa
H3
15kDa

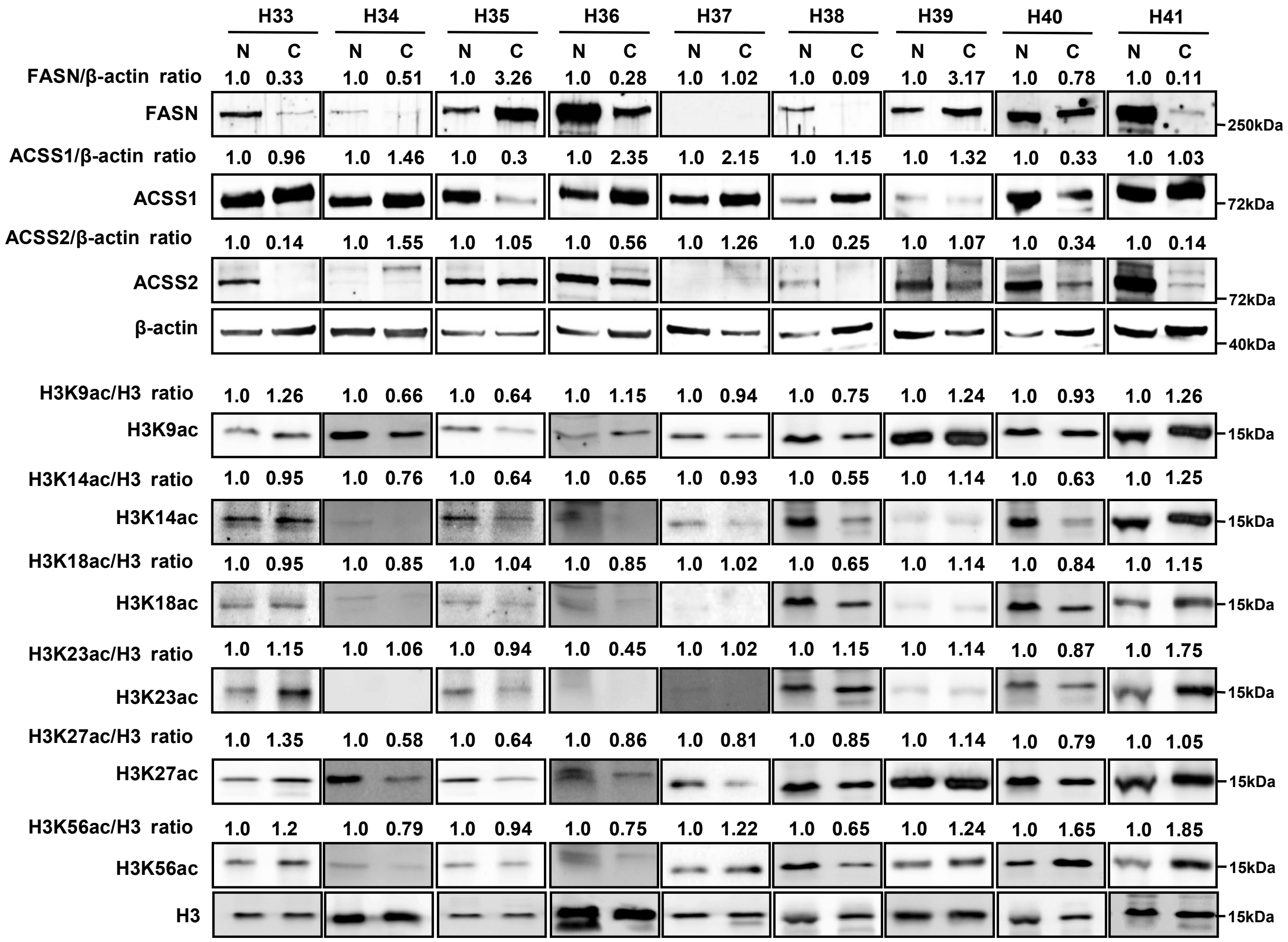

H33 H34 H35 H36 H37 H38 H39 H40 H41
N C N C N C N C N C N C N C N C N C
FASN/β-actin ratio 1.0 0.33 1.0 0.51 1.0 3.26 1.0 0.28 1.0 1.02 1.0 0.09 1.0 3.17 1.0 0.78 1.0 0.11
FASN
250kDa
ACSS1/β-actin ratio 1.0 0.96 1.0 1.46 1.0 0.3 1.0 2.35 1.0 2.15 1.0 1.15 1.0 1.32 1.0 0.33 1.0 1.03
ACSS1
72kDa
ACSS2/β-actin ratio 1.0 0.14 1.0 1.55 1.0 1.05 1.0 0.56 1.0 1.26 1.0 0.25 1.0 1.07 1.0 0.34 1.0 0.14
ACSS2
72kDa
β-actin
40kDa
H3K9ac/H3 ratio 1.0 1.26 1.0 0.66 1.0 0.64 1.0 1.15 1.0 0.94 1.0 0.75 1.0 1.24 1.0 0.93 1.0 1.26
H3K9ac
15kDa
H3K14ac/H3 ratio 1.0 0.95 1.0 0.76 1.0 0.64 1.0 0.65 1.0 0.93 1.0 0.55 1.0 1.14 1.0 0.63 1.0 1.25
H3K14ac
15kDa
H3K18ac/H3 ratio 1.0 0.95 1.0 0.85 1.0 1.04 1.0 0.85 1.0 1.02 1.0 0.65 1.0 1.14 1.0 0.84 1.0 1.15
H3K18ac
15kDa
H3K23ac/H3 ratio 1.0 1.15 1.0 1.06 1.0 0.94 1.0 0.45 1.0 1.02 1.0 1.15 1.0 1.14 1.0 0.87 1.0 1.75
H3K23ac
15kDa
H3K27ac/H3 ratio 1.0 1.35 1.0 0.58 1.0 0.64 1.0 0.86 1.0 0.81 1.0 0.85 1.0 1.14 1.0 0.79 1.0 1.05
H3K27ac
15kDa
H3K56ac/H3 ratio 1.0 1.2 1.0 0.79 1.0 0.94 1.0 0.75 1.0 1.22 1.0 0.65 1.0 1.24 1.0 1.65 1.0 1.85
H3K56ac
15kDa
H3
15kDa

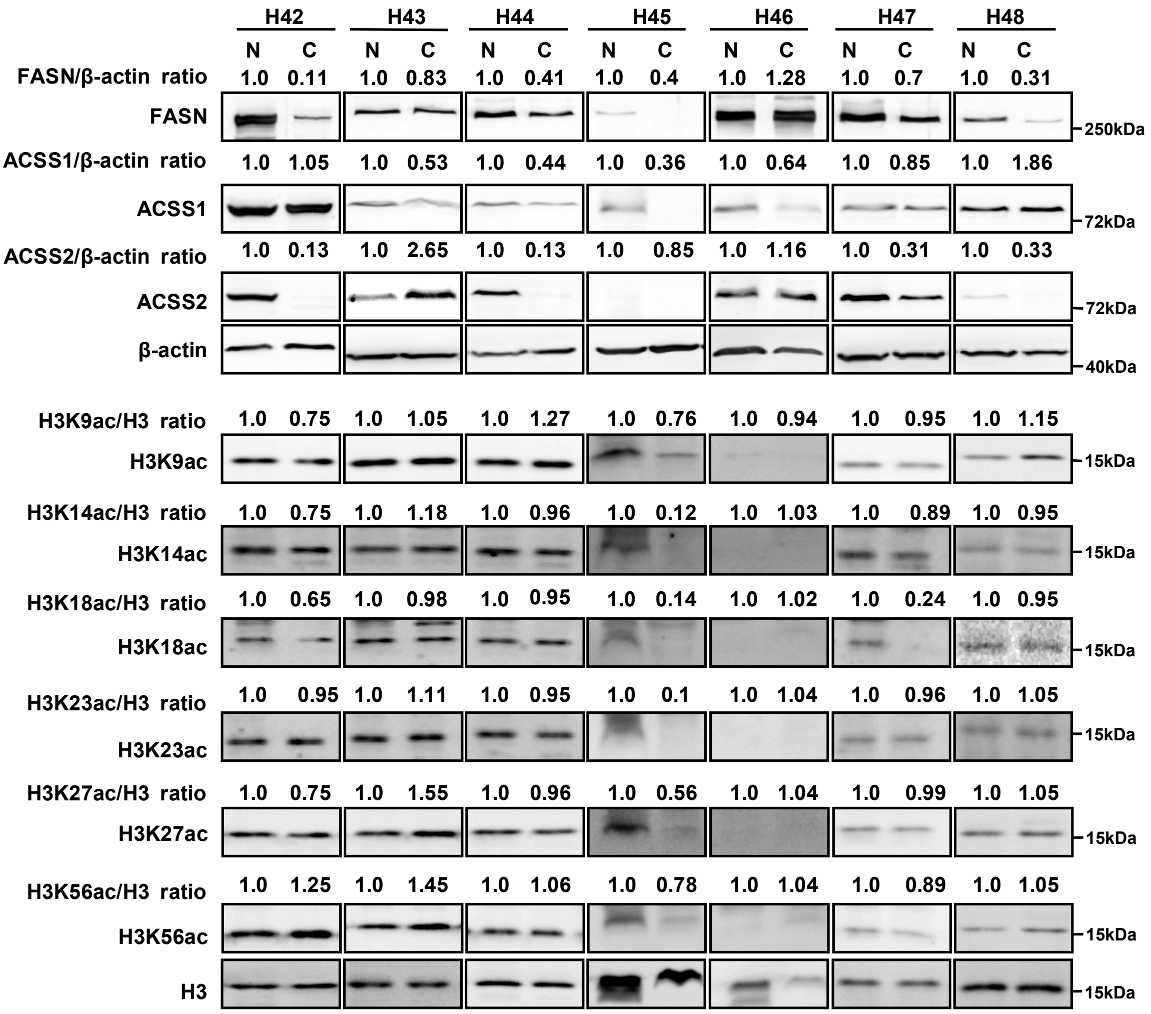

H42 H43 H44 H45 H46 H47 H48
N C N C N C N C N C N C N C
FASN/β-actin ratio 1.0 0.11 1.0 0.83 1.0 0.41 1.0 0.4 1.0 1.28 1.0 0.7 1.0 0.31
FASN
250kDa
ACSS1/β-actin ratio 1.0 1.05 1.0 0.53 1.0 0.44 1.0 0.36 1.0 0.64 1.0 0.85 1.0 1.86
ACSS1
72kDa
ACSS2/β-actin ratio 1.0 0.13 1.0 2.65 1.0 0.13 1.0 0.85 1.0 1.16 1.0 0.31 1.0 0.33
ACSS2
72kDa
β-actin
40kDa
H3K9ac/H3 ratio 1.0 0.75 1.0 1.05 1.0 1.27 1.0 0.76 1.0 0.94 1.0 0.95 1.0 1.15
H3K9ac
15kDa
H3K14ac/H3 ratio 1.0 0.75 1.0 1.18 1.0 0.96 1.0 0.12 1.0 1.03 1.0 0.89 1.0 0.95
H3K14ac
15kDa
H3K18ac/H3 ratio 1.0 0.65 1.0 0.98 1.0 0.95 1.0 0.14 1.0 1.02 1.0 0.24 1.0 0.95
H3K18ac
15kDa
H3K23ac/H3 ratio 1.0 0.95 1.0 1.11 1.0 0.95 1.0 0.1 1.0 1.04 1.0 0.96 1.0 1.05
H3K23ac
15kDa
H3K27ac/H3 ratio 1.0 0.75 1.0 1.55 1.0 0.96 1.0 0.56 1.0 1.04 1.0 0.99 1.0 1.05
H3K27ac
15kDa
H3K56ac/H3 ratio 1.0 1.25 1.0 1.45 1.0 1.06 1.0 0.78 1.0 1.04 1.0 0.89 1.0 1.05
H3K56ac
15kDa
H3
15kDa

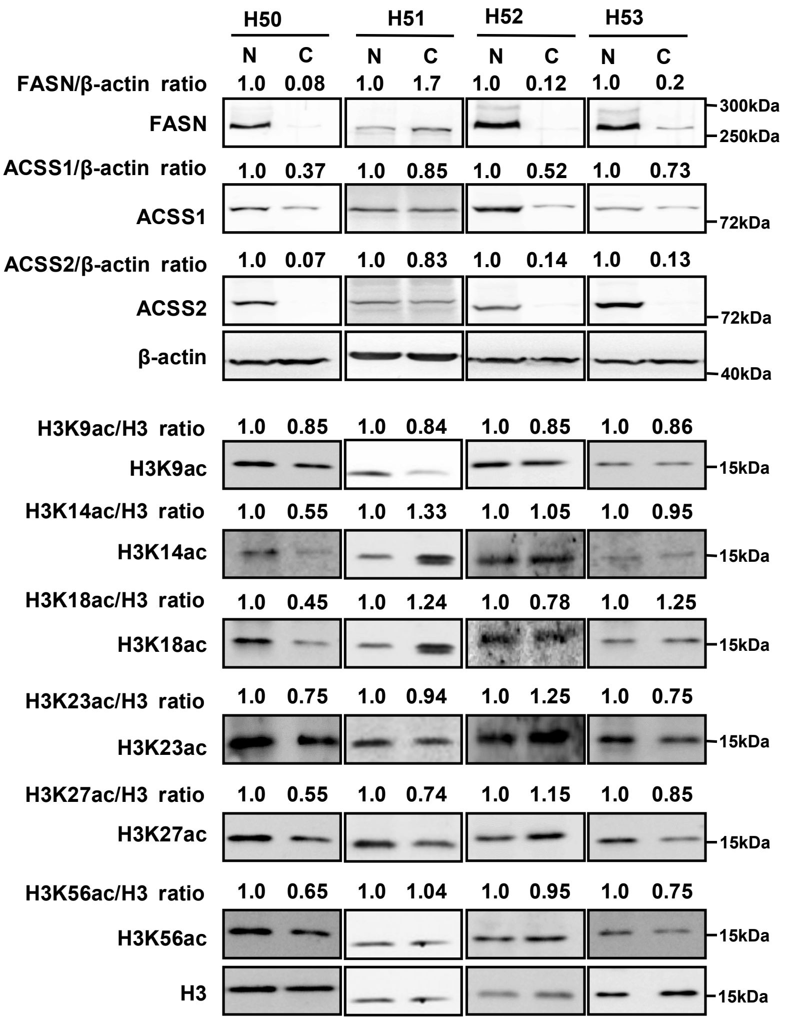
H50
H51
H52
H53
N C N C N C N C
FASN/β-actin ratio 1.0 0.08 1.0 1.7 1.0 0.12 1.0 0.2
FASN
300kDa
250kDa
ACSS1/β-actin ratio 1.0 0.37 1.0 0.85 1.0 0.52 1.0 0.73
ACSS1
72kDa
ACSS2/β-actin ratio 1.0 0.07 1.0 0.83 1.0 0.14 1.0 0.13
ACSS2
72kDa
β-actin
40kDa
H3K9ac/H3 ratio 1.0 0.85 1.0 0.84 1.0 0.85 1.0 0.86
H3K9ac
15kDa
H3K14ac/H3 ratio 1.0 0.55 1.0 1.33 1.0 1.05 1.0 0.95
H3K14ac
15kDa
H3K18ac/H3 ratio 1.0 0.45 1.0 1.24 1.0 0.78 1.0 1.25
H3K18ac
15kDa
H3K23ac/H3 ratio 1.0 0.75 1.0 0.94 1.0 1.25 1.0 0.75
H3K23ac
15kDa
H3K27ac/H3 ratio 1.0 0.55 1.0 0.74 1.0 1.15 1.0 0.85
H3K27ac
15kDa
H3K56ac/H3 ratio 1.0 0.65 1.0 1.04 1.0 0.95 1.0 0.75
H3K56ac
15kDa
H3
15kDa

c

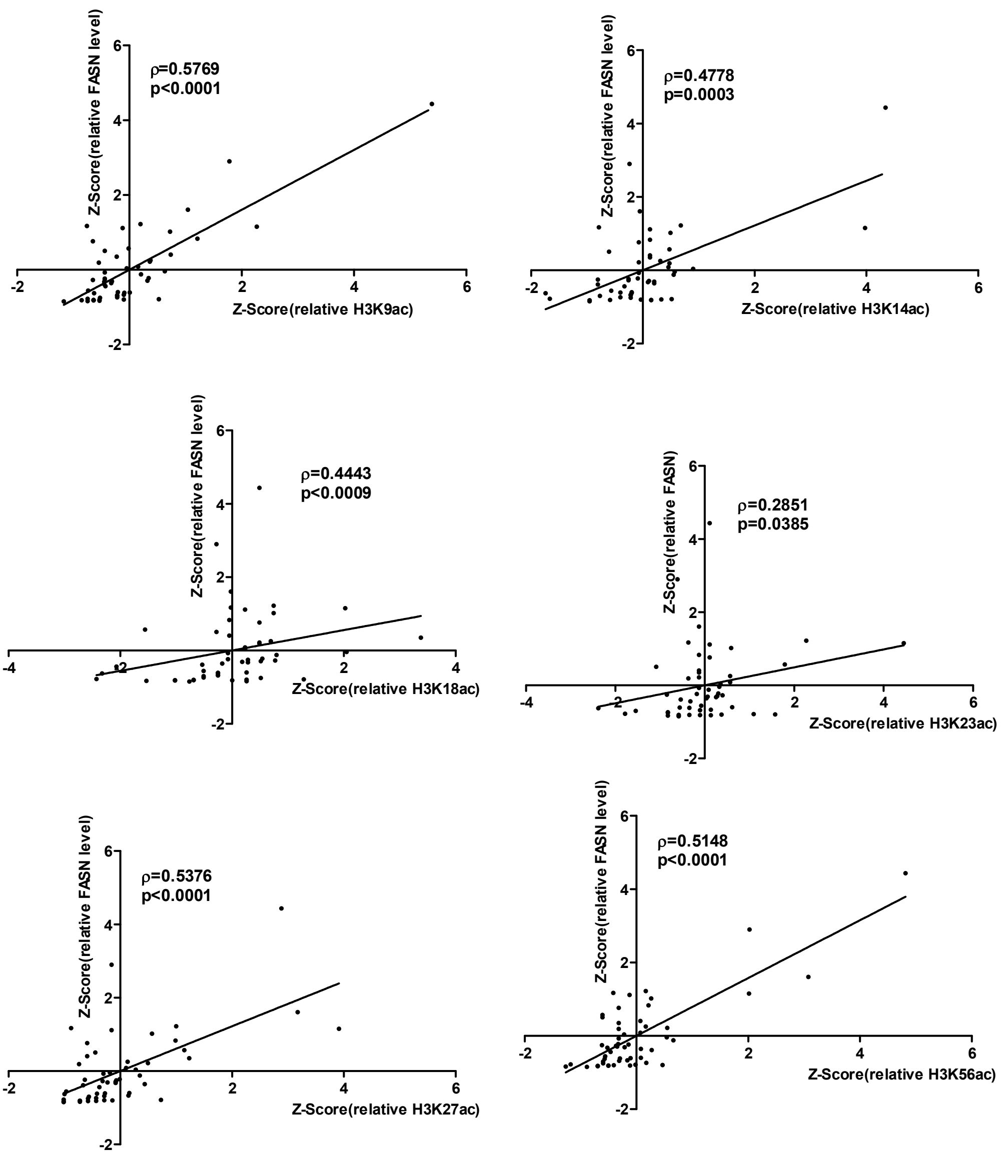

Supplementary Figure 6 ACSS1/2 positively correlates with histone H3 acetylation marks and FASN expression in human hepatocellular carcinoma

(**a**) Correlations between *FASN* and *ACSS1/2* mRNA levels in 190 human hepatocellular carcinoma samples are assessed by incorporating data from TCGA. Significance of correlation is presented as Pearson's coefficient. (p= -0.096 for *ACSS1* v.s. *FASN*, p=0.51 for *ACSS2* v.s. *FASN*). (**b**) Western blot analysis of FASN, ACSS1 and ACSS2 protein levels (upper) and 6 histone H3 acetylation marks (lower) in human hepatocellular carcinoma (n=51). (**c**) Histone H3 acetylation marks exhibit significantly positive correlation with FASN expression ($p<0.0001$ for H3K9ac, $p=0.0003$ for H3K14ac, $p=0.0009$ for H3K18ac, $p=0.0385$ for H3K23ac, $p<0.0001$ for H3K27ac, and $p<0.0001$ for H3K56ac). Spearman's correlation was analyzed between histone acetylation and FASN expression in all 53 samples.

Supplementary Figure 7 Full Scans of Western Blotting Data in Main Figures

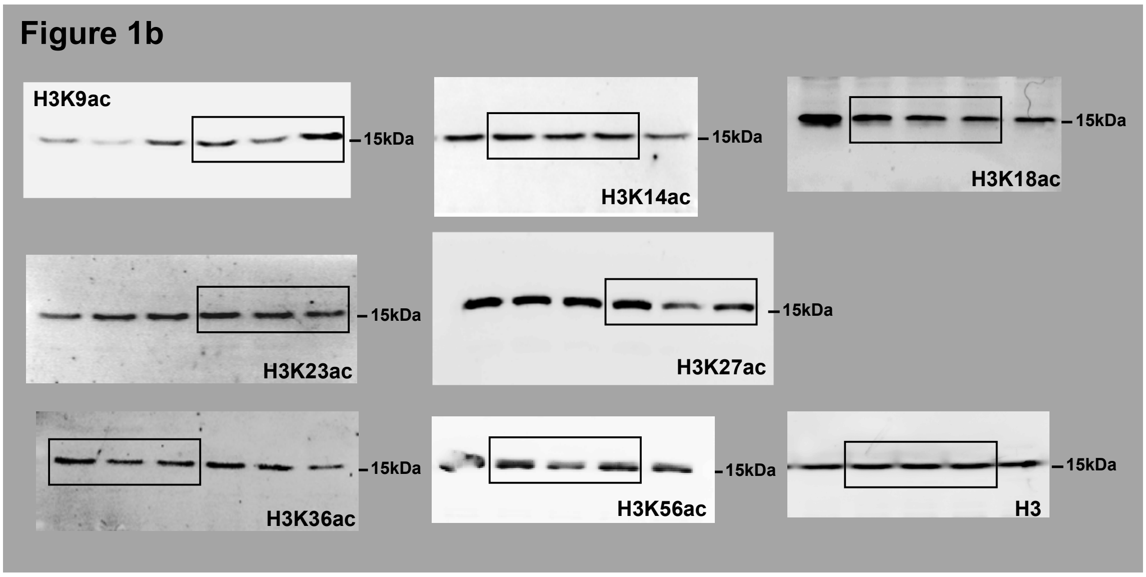

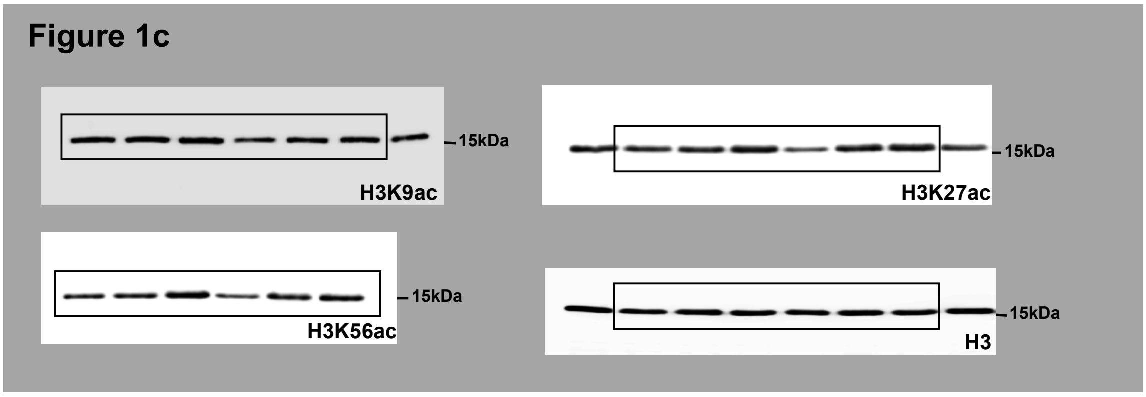

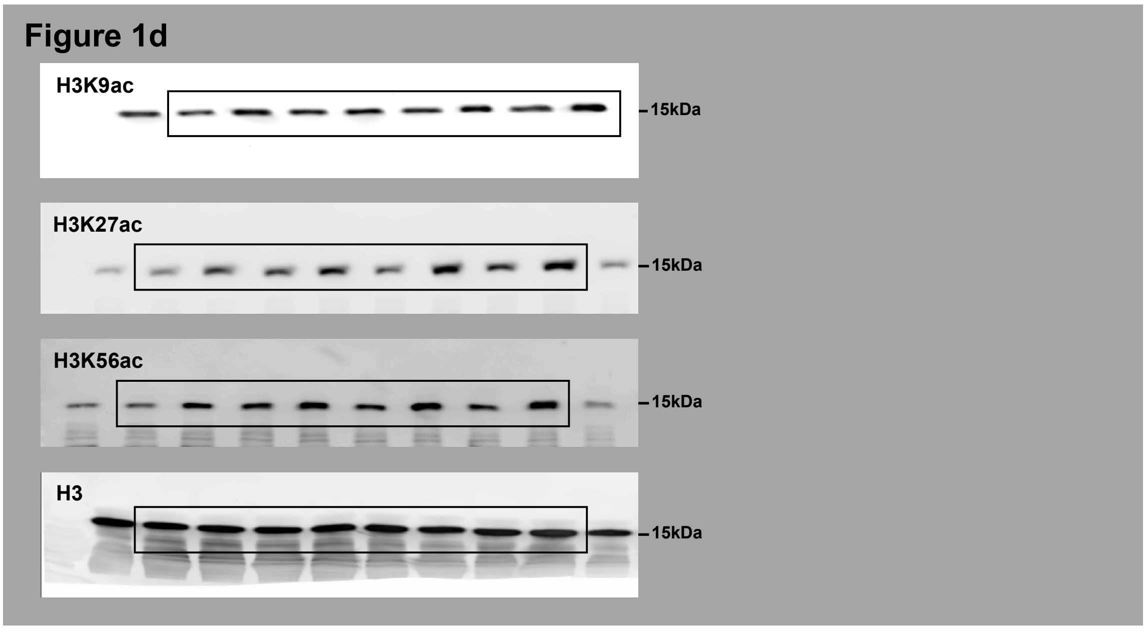

Figure 1e

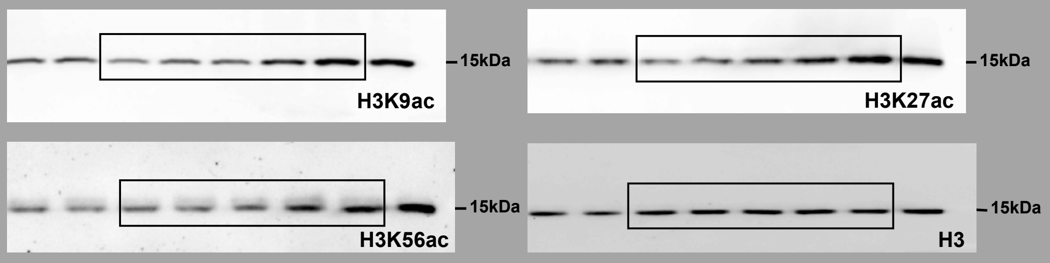

Figure 4a

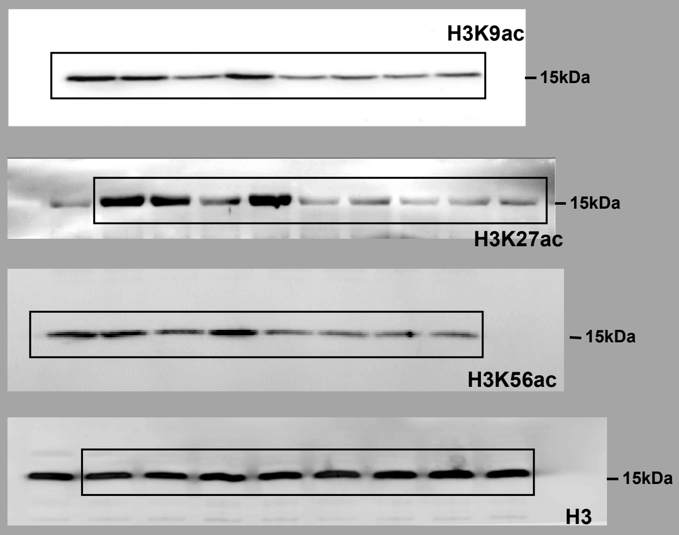

Figure 6a

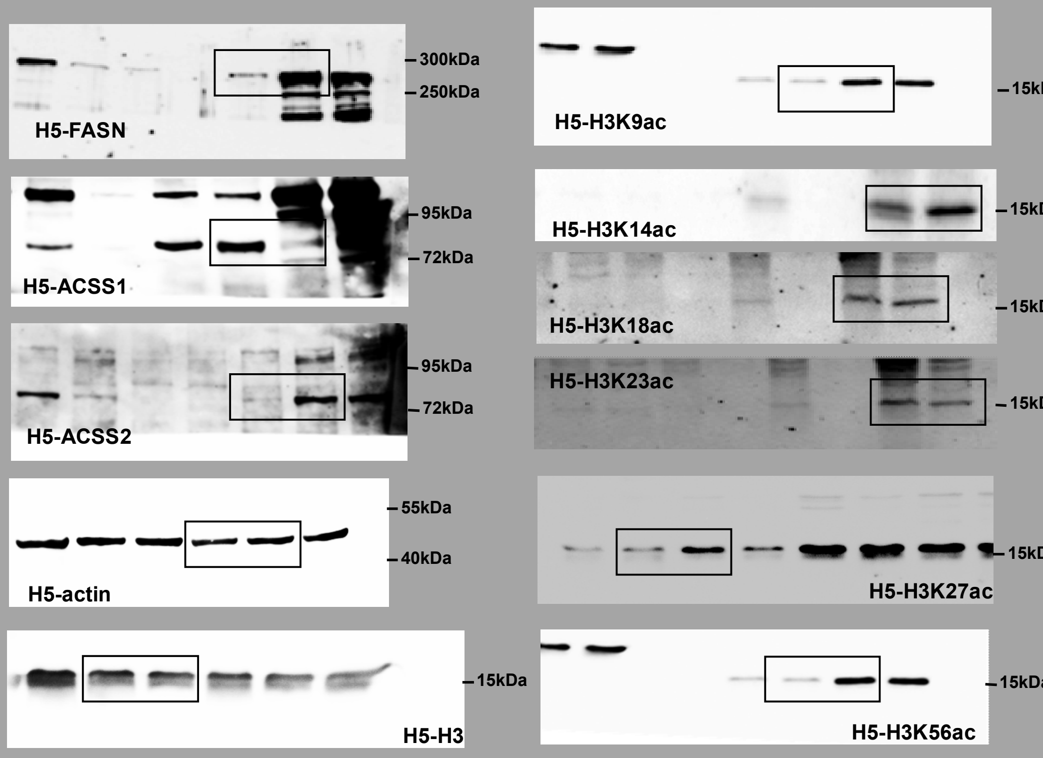

Figure 6a

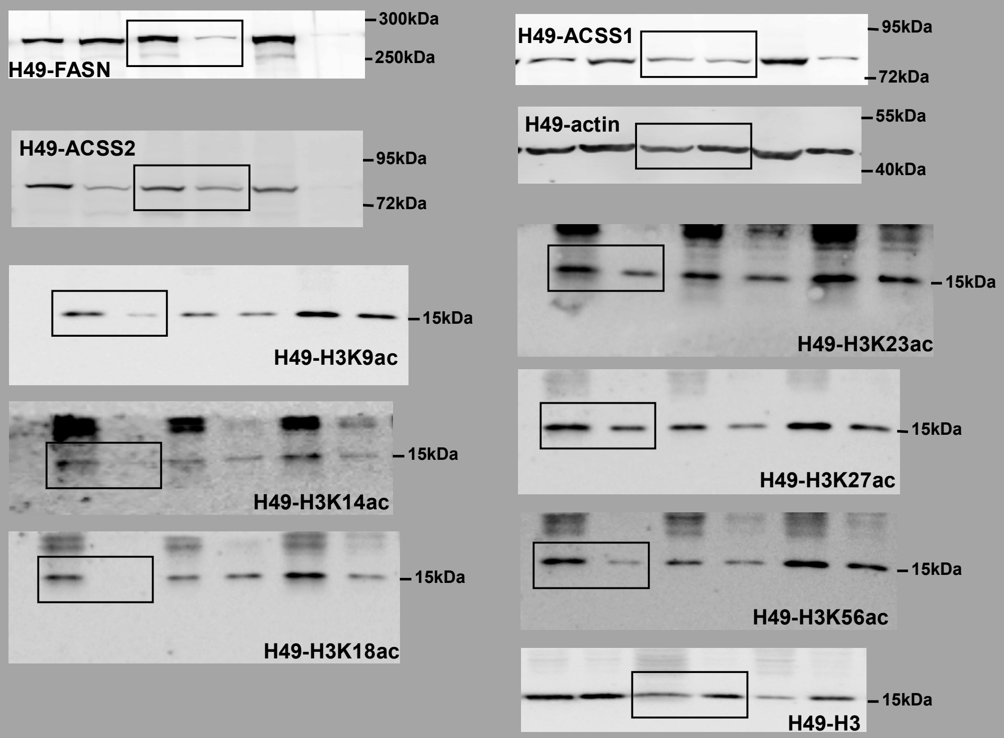
H49-FASN
300kDa
250kDa
H49-ACSS1
95kDa
72kDa
H49-ACSS2
95kDa
72kDa
H49-actin
55kDa
40kDa
H49-H3K9ac
15kDa
H49-H3K23ac
15kDa
H49-H3K14ac
15kDa
H49-H3K27ac
15kDa
H49-H3K18ac
15kDa
H49-H3K56ac
15kDa
H49-H3
15kDa

Supplementary Table 1 Primers used in this study

Metabolic pathways	Gene	Forward primer (5` to 3`)	Reverse primer (5` to 3`)
Lipid synthesis pathway	*ACACA*	AGTGGGTCACCCCATTGTT	TTCTAACAGGAGCTGGAGCC
	FASN	GAAGCTCGTGTTGACTTCTC	AGAAGACCACAAAGTAGTCC
	ACSS1	ACCAAGATCGCCAAATATGC	TGCTTGTCCTTGCACTTCTG
	ACSS2	GGATTCCAGCTGCAGTCTTC	CATGCCACCACAAGTCAATC
	ACLY	GGTGCTCCGGATTTTGC	ACATGGCTGCAGAGAGACCT
	SCD1	GCAGCCGAGCTTTGTAAGAG	GTTCTACACCTGGCTTTGGG
Glycolysis pathway	*ALDOA*	GAACACACCCCCTCAGCCCTC	CAAGCATGGCCTGGGGTGA
	ENO1	TACGTTCACCTCGGTGTCTG	CCTGGCATGGATCTTGAGAA
	LDHA	GGAGATCCATCATCTCTCCC	GGCCTGTGCCATCAGTATCT
	BPGM	GGCACTCCTAAAACACCTGG	CGCAGGTTTTCATCCAATTC
	GPI	GCTCGAAGTTGTCAAAACCC	AAACATGTTCGAGTTCTGGGA
	TPI1	TATAGGCAGTAGGGGGAGCA	GAAGCAGAGTCTGGGGGAG
	PKM2	GTCTGAATGAAGGCAGTCCC	TCCGGATCTCTTCGTCTTTG
	GAPDH	AATGAAGGGGTCATTGATGG	AAGGTGAAGGTCGGAGTCAA
	ALDH8A1	AAATTTGGGCACCTTCAGC	TCCACTGGTGAGCATAGGTG
	HK2	AGCCCTTTCTCCATCTCCTT	AACCATGACCAAGTGCAGAA
TCA cycle	*FH*	ATCCTGGTTTACTTCAGCGG	GTATTATGGCGCCCAGACC
	IDH1	GGCTTGTGAGTGGATGGGTA	TCCGTCACTTGGTGTGTAGG
	CLYBL	GGGGATATCAGCTGCTAGAGAC	GAAGATGGCGCTACGTCTG
	ACO1	CAATGGCTCAGCAAGGTGT	CTGCTTGGGTCAGGTTCG
	IDH2	TACGGGTCATCTCATCACCA	ACCTCGCAAGAGCAGCC
	IDH3A	CCTCTGGTCACCTGTTTTGG	CAGGAGGGGAAGCGATG
	PDK1	ATTTTCCTCAAAGGAACGCC	CAACAGAGGTGTTTACCCCC
PPP pathway	*G6PD*	CACCAGATGGTGGGGTAGAT	AGAGCTTTTCCAGGGCGAT
	RPE	GGCTTTACCCACTGTTCTGG	GGATGCTAGACTCTGGGGC
	RPIA	CGATCCAGATCACTGAGGGT	GCTGAAAGGGTGAAGCAAGA
	TALDO1	ATCCTGGGGCTTGTACTCGT	GAAGCGTCAGAGGATGGAGT
	TKT	GCATGGTGTGGAAAAAGAGG	CGCCTACGTATCAGCTCCA
Glycogen metabolism	*GBE1*	CCACCTTCATTTTCTCCAATG	CCAGACTCCTGGAGATCGAC
	AGL	ACGGTAACTGCTTTTCCCTG	TGAACGAAATGGAGAAACTGG
	PGM1	GAGGATTCCATTCTGTCCGA	CCGGTTCTACATGAAGGAGG
	PGM2	AGCACTGACCAGTTCATTCAG	GGACAGAGCCCAAAATCAAG
	PGM3	ATGTAGTGCAGCTGGGGTGT	GGCCCAGCAGTGAGAAACT
	PYGL	GGGTTTCCATATCTGAGCCA	AGCCTATGGATACGGCATTC
Ketogenesis & ketone body metabolism	*HMGCS1*	TGGCAGGGAGTCTTGGTACT	TCCCACTCCAAATGATGACA
Amino acid metabolism	*GOT2*	GGATCTGGAGGTCCCATTTC	ATGGCCCTGCTGCACTC
	MAOB	GGCTTCCAGAACAACCACAT	CATGAGCAACAAATGCGAC
	ACAT1	TTGATACATAACTCCGTTCCACA	GACCATGGCTGTGCTGG

	GLS	GCAAATCTTCGAAGTGCAGAC	AAAAACTTGATCCTCGAAGAGA
	BCAT2	GCTGGTCTTTGCCTTTGAAC	CCTTCCAGAACCTCACGCT
	SHMT2	TTTGCTTCCCCAGTCTGAGT	TTCTCTTTGTTTTGGGCGG
	GLDC	AGCCCATGCCAATATACGAT	CCCTTGAAAATGGAAGACCC
	ACY1	GGGCTGTCTCCTCAAAGAAA	CATCGGTGACGCTCTTCC
	AGMAT	CACTGGGCCATGCTTTTT	AGCAGCTGGCTGTATTCCTC
	ALDH18A1	ATAAACGGGATGTTGCTCCA	AACCAACATCTTCTGCCCTG
	ALDH9A1	CTTGCCATTGTTGATGCACT	TCAAAAATCTGGCATGGAGC
	AMD1	CCTGCTTGTCAGTTTTTGTCAC	GGATCTTCGCACTATCCCAA
	ARG2	GATCATCTTTGGGGACTGGA	CTGCCATAAGAGAAGCTGGC
	ASL	GACGCGTTGAACTTCTCCAT	AAGCTTCCGGACGACGA
	ASS1/ass	GCCTCGATGTCTAAATGAGCA	ATTGACATCGTGGAGAACCG
	CKB	CCCACACCAGGAAGGTCTTA	CCTCTTCGACAAGCCCGT
	CPS1	CCCAAGGCATTTTGAAATCT	GGAAAAGACACTGAAAGGGCT
	GATM	TAATGCCCTCCTTGCTTCTG	AGAAAACGCCTGTGTTCCAC
	GLUD1	CTCACATCAGTGCTGTAACGG	GGCTCCTGGGAGGTCATC
	NAGS	GTTGGAGAAGCTGCCATCAC	CGGGACCTTCAGACACTTTT
	NOS2	TCATTCTGCTGCTTGCTGAG	CAACAATGTGGAGAAAGCCC
	OAT	AAGTACAGCAAACCTCTGCAAAT	AATTCCTGTCCTCAGGCG
	OTC	AATACTGCATCTGCCATGCT	CTTCTGGGAGGACATCCTTG
	SAT1/sat	ATGGCAAAACCAACAATGCT	TTTGGAGAGCACCCCTTTTA
	SRM	CACATGTAGGGTCACCTTCG	TGGTCCAGTGTGAGATCGAC
	CTH	AATATCAGCTCCCAGAGCCA	TTGTCCATAAGCATGGAGACAT
	DAO	GAGTGGTAGCGCTCATGGAT	CCAGCAGTTTGGTACTTCCG
	LAP3	ATTTGCAGAAGCCTTGATGG	ACTACAAAGGCAGCCCCAAT
	ODC1	CCCTTGGAACAGCAGTGAC	GGCTGCGACTCAGGCTC
	P4HA1	CAAAGACTGGGGAAGCAGAA	CCTCTCGTCCCACTTTCCA
	PRODH	ATTGCTTGTCCCGCTTATTC	GGAGTGGAGGAGGACCTGA
	PRODH2	AGCATTGAGGCAGGAGACCT	CTAGCCTCGTGGGTCAGAAG
	PYCR	CACCTTGTCCAGGATGGTCT	CTCCTGCATCCGCACAC
	AADAT	GTGGGTGCAGACTTTGAAGAG	GGCAGCCAACAAGGTCTTT
	ACMS	GGTTCCAGCTCACCTAGTGG	TGATCCTCTGTCCCTCAAGC
	MAT1A	GCACTGCATCACTGATCTGG	TGGCTTGTGTGACCACTCTC
	MTAP	TGGCACAAGAATGACTTCCA	CTTGAGGGAGGAGATTCAGC
	MTR	TTCACAAGCAGATGGTAGGC	GCTATGGTGGTCATGGCTTT
	ABAT	CTTGAGGGACAGGGACACC	ATCCCGCAAGTCGGAAC
	ADSL	AACGTTTCCCAACTGTGGTC	AGAGTGATCTCTCGGCTTGC
	GOT1	ATTCGAAGTGCACCTGTTCC	AGCTGTGCTTCTCGTCTTGC
	AGXT	GCCCATGCAGATACGCC	ACAGCCTGAGAGAGAGCCTG
	ALAS1	AGCTGTGTGCCATCTGGACT	AGATCAAAGAAACCCCTCCG
	AMT	CTCCAGCCTCGTTGGTAAAC	GTAGTGACCGGGTGAAGCTG
	BHMT	GCAGTTCACACCAATGATGG	ATTGGCCCAGAAGGAGATTT
	CHDH	ACGGAGGGGAATGAGATGAC	CCCGGACAATCACAAAGAGT

	DAO	GAGTGGTAGCGCTCATGGAT	CCAGCAGTTTGGTACTTCCG
	DLD	CCAACTCCACCTACATGACCT	TGGTTGTTATTGGTGCAGGA
	GCAT	ACAGCTGGGATAGAGGATGG	GGTCCGCTTTATCTGTGGAA
	GLDC	AGCCCATGCCAATATACGAT	CCCTTGAAAATGGAAGACCC
	MAOA	CAGTCAAGAGTTTGGCAGCA	TGGAGAATCAAGAGAAGGCG
	PHGDH	TTCTCAGCTGCGTTGATGAC	GCAAAGAGGAGCTGATAGCG
	PIPOX	TGCTGTGATGACCAAGCTCT	CATAGTGCGTGACGGAGAGA
	PSAT1	TATACAGAGAGGCCCGGATG	GGAGAAGCTTAGCTCCATCAAA
	MATIIa	GCATCAAGGACAGCATCACT	AGGAGGGCACATTCCTTTTC
	PSPH	CCAGGGAGGTGAGCTGTG	AGTCATTCCTGCCTTTGAGC
	GLS2	AAAAGAGCAAATCACCCAGG	CAGAGAGACGCCACACAGC
	SHMT2	TTTGCTTCCCCAGTCTGAGT	TTCTCTTTGTTTTGGGCGG
Fatty acid beta-oxidation	*HSD17B4*	GAAGTCCCCTCCCAAATCAT	GTGGTACTGGTCACCGGC
	ECHS1	TTTTCTGCGATGATGTACTCAAA	CTGCGTGTCCTGCTGTCCT
	EHHADH	CTGAATTGGCTTGTTGCAGA	CTCAGACCCGGTTGAAGAAG
	ACADS	CCCATCTTCTTCACCTGAGC	AGATGTTGCTCCAGACATGC
	ACAA1	CACTCAGAAACTGGGCGATT	CTCAAGGACGTGAATCTGAGG
	CPT1A	GCCTCGTATGTGAGGCAAAA	TCATCAAGAAATGTCGCACG
	ACADL	TCATGCAGCTGGAGACAGTT	TTGGCAAAACAGTTGCTCAC
	HADHSC	ACTGTGTGACCAGTTGCTGC	GCCTCGGCCAAGAAGATAAT
	ACACB	CAGCTTCTTGTGTTCCCGTC	CCTGGAGGCTTATCTGACCA
	HADHA	CCGTTCCTCTGGAGGTTTTA	TGGTAGAAGCATTCGTGCAG
	CPT2	CGGAGTCTCGAGCAGATAGG	GGAAAAGAACTGCATGAGCA
Gluconeogenesis pathway	*PEPCK1*	ATGCCGATCTTTGACAGAGG	GAGAAAGCGTTCAATGCCAG
Cholesterol synthesis pathway	*HMGCS1*	TGGCAGGGAGTCTTGGTACT	TCCCACTCCAAATGATGACA
	CYP7A1	GGTGCAAAGTGAAATCCTCC	CAGAACTGAATGACCTGCCA
	HMGCR	GATGGGAGGCCACAAAGAG	TTCGGTGGCCTCTAGTGAGA
	SOAT1	ATTCCTCTGCCTCTGCTGTC	AGACCAGAGAAACCCTGCAA
	FDFT1	TCGGCAATCACTGTTTGGTA	GGTTCATGGAGAGCAAGGAG
	SQLE	CAACAGTCATTCCTCCACCA	AGCAAGCTTCCTTCCTCCTT
	DHCR24	CGAAGAGGTAGCGGAAGATG	ACTACTACCACCGACACACGC
	LSS	AAACATTCAGGACAGCCAGC	GCCTGATGATCCTGACCTTG
	MVD	TCGTTTTTAGCTGGTCCTGG	CGGTCAACATCGCAGTTATC
	FDPS	ATACCAGCAGATCTGTCCCC	TGGGCTGGTGTGTAGAACTG
Steatolysis	*GPD1*	CTTGGTGTTGTCACCGAAGC	CAAGAGGTGGACACAGTGGA
	AGPAT5	ACTGTGCTTTGATGATTCGCT	TCTTCGAGAACTACACCGGG
	CDIPT1	GACCAAGAGACACATGGTGG	CTAGACGCTTTCGATGGACAC
	PLD2	CTGCCACAGCAGCAAAGTAA	CAGCTACATCAGCATGACAGC
	PPAP2A	ACAGAAAAAGCCCCTCTGAAA	CTCGATGTGATTTGCGTGTT
	PPAP2B	CTTGATGCTCTCGTCATTGC	CCTCTTCTGCCTCTTCATGG
	ATGL	ACCTCAATGAACTTGGCACC	CAACGCCACGCACATCTA

Sphingolipid metabolism	*SPTLC2*	CAGGCGTCTCCTGAAATACC	TCACCTCCATGAAGTGCATC
	DEGS1	TGGTCAGGTTTCATCAAGGAC	AATGGGTCTACACGGACCAG
Nucleotide metabolism	*MTHFD2*	CACTCTTCTACCTCCTGCCG	TTCGCCCTTTCCACCTC
	SRM	CACATGTAGGGTCACCTTCG	TGGTCCAGTGTGAGATCGAC
	ADSL	AACGTTTCCCAACTGTGGTC	AGAGTGATCTCTCGGCTTGC
	PDE8B	GAATAGGGTCCTGCGTCAGT	GCCGAGACTCAGACCTGCTA
	MTHFD1	TTGATTTTTCAGTCTCGCCC	TATTGGTGGTGTCCATCGTG
Transctiption factor	*SREBF1*	CTGGTCTACCATAAGCTGCAC	GACTGGTCTTCACTCTCAATG
	SREBF2	TCAGGGAACTCTCCCACTTG	GAGACCATGGAGACCCTCAC
	PGC1 α	CTGCTAGCAAGTTTGCCTCA	AGTGGTGCAGTGACCAATCA
	PRKCA	CAAATTCATGGCACCTCTTG	CACTGCACCGACTTCATCTG
	MYC	CACCGAGTCGTAGTCGAGGT	TTTCGGGTAGTGGAAAACCA
Glucose transporter	*GLUT1*	TCTGGCATCAACGCTGTCTTC	CGATACCGGAGCCAATGGT
	GLUT4	AGCACCGCAGAGAACACAG	GTCGGGCTTCCAACAGATAG
	SLC2A1	GGCATTGATGACTCCAGTGTT	ATGGAGCCCAGCAGCAA
Acetate transporter	*SLC16A1*	AACTGGACCTCCAACTGCTG	GCGATCCGCGCATATAAC
	SLC16A4	AAGAGCCATTGTATGTCTGGG	GGGACTTGCCAGTTTCTTTG
Amino acid transporter	*SLC1A5*	GGGCAAAGAGTAAACCCACA	CACCATGGTTCTGGTCTCCT
Positive control	*VEGF*	CTCGATTGGATGGCAGTAGCT	AGGAGGAGGGCAGAATCATCA
	ACTB	GCACAGAGCCTCGCCTT	GTTGTCGACGACGAGCG

Sequences of primers targeting 139 different metabolic genes for RT-PCR

Gene	Forward primer (5\` to 3\`)	Reverse primer (5\` to 3\`)
FASN	GATAGCCTATGCTCTGGGGG	CTCCTGTGGTGTGTGGGTTG
ACACA	TGATTGAAACGCACCCTC	ACGTTCCCATCTCCACCC
ACLY	TTGTACCCACGGAACCAA	CTCATCGGGACGCCTTTT
LDHA-a	CGTCGAGTTTTGGAGGTCAC	ATCCATGAAACCTGGGGAGG
LDHA-b	TACTGGTGTATCTCGGGCTG	TCCCTGACTGTCTCCTAGCT
VEGF-a	GAAAATTACCCATCCGCCCC	GATCTGTGTGTCCCTCTCCC
VEGF-b	TTTTGCTTGCCATTCCCCAC	CCCAAAAGCAGGTCACTCAC

Sequences of primers targeting *FASN*, *ACACA*, *ACLY*, *LDHA*, and *VEGF* promoter regions in ChIP-qPCR assay
